# Ethno-medicinal uses of vertebrates in the Chitwan-Annapurna Landscape, central Nepal

**DOI:** 10.1371/journal.pone.0240555

**Published:** 2020-10-30

**Authors:** Jagan Nath Adhikari, Bishnu Prasad Bhattarai, Maan Bahadur Rokaya, Tej Bahadur Thapa

**Affiliations:** 1 Central Department of Zoology, Institute of Science and Technology Tribhuvan University, Kirtipur, Kathmandu, Nepal; 2 Department of Zoology, Birendra Multiple Campus, Tribhuvan University, Bharatpur, Chitwan, Nepal; 3 Institute of Botany, Czech Academy of Sciences, Průhonice, Czech Republic; 4 Department of Biodiversity Research, Global Change Research Institute, Czech Academy of Sciences, Brno, Czech Republic; Universidad Mayor de San Andrés, PLURINATIONAL STATE OF BOLIVIA

## Abstract

Traditional knowledge on the use of animal products to maintain human health is important since time immemorial. Although a few studies reported food and medicinal values of different animals, a comprehensive ethno-medicinal study of vertebrates in Nepal is still lacking. Thus, present study is aimed at documenting the ethno-medicinal knowledge related to vertebrate fauna among different ethnic communities in the Chitwan-Annapurna Landscape, central Nepal. Data was collected by using semi-structured questionnaires and analyzed by using Use Value (UV), Informant Consensus Factor (ICF) and Fidelity level (FL). Results showed a total of 58 (53 wild and 5 domestic) species of vertebrate animals. They were used to treat 62 types human ailments. Four animals were also used for veterinary diseases and agriculture benefits. The most widely used species was *Felis chaus* (UV = 0.25) with 3 use-reports by 10 informants. Cardiovascular and dental problems had the highest ICF value (0.974) with cardiovascular problems having 351 use-reports for 10 animal species and dental problems having 77 use-reports for 3 animal species. The least ICF was found in ophthalmological problems (ICF = 0.833, use reports = 7 for 2 species). We concluded that the different animals were an important part of traditional medicine for the local people living in the Chitwan-Annapurna Landscape. However, the majority of animals and most likely to be threatened due to their uses. The present documented ethnozoological knowledge can be used in conservation and management of vertebrates so that they could be protected for future generations.

## Introduction

Bio-resources, both flora and fauna, are integral part of the indigenous healing practices used by human beings since the prehistoric time [1–4]. The traditional knowledge on the use of bio-resources for medicine has a significant contribution in maintaining the human health [3, 5–8]. In traditional medicine, it is estimated that more than 60% of medicines is based on flora and fauna [9, 10] and different chemical compounds derived from plants and animals are used to improve human health [11, 12]. The World Health Organization (WHO) has selected a total of 252 essential chemical compounds to prepare drugs, where animal based compounds contribute about 8.7% [9]. A figure shows the use of more than 1500 animal species in the Traditional Chinese Medicine in China [13] and more than 100 animal species in Nepal [4, 14–18]. In addition to the use of animals as medicine, many animals are also traded for various purposes in the world [19–21].

Animals have significantly contributed as therapeutic agents in preventing and treating several human ailments or disorders across the globe [10, 22–28]. Zootherapy is considered as an important alternative therapeutic practice [29–31]. Studying about zootherapy allows us to understand different aspects of social and cultural systems of indigenous people and also find out their inter-relationships between people and animals [29, 32]. In addition to this, investigating the use of animals in traditional medicine could highlight an important aspect of indigenous knowledge [27, 33]. Recent studies have shown that there is a loss of many animal species [34, 35] as they are used in different traditional systems such as Ayurveda, Unani, Homeopathy, Chinese and Tibetan traditional medicines [9, 19, 36, 37]. Animals origin medicine cover about 15–20% of the Ayurvedic medicines [27]. Studies in Nepal have shown the use of animals as medicine [4, 15, 36, 38–42] including a few studies from the Chitwan-Annapurna Landscape (CHAL), but are mainly related to medicinal plants [37, 43–46] and wild edible plants [47]. The Chitwan-Annapurna Landscape lies in between the Chitwan National Park in the south and the Annapurna Conservation Area in the north, ranges from 150m to 8000m above sea level (m asl). This area is inhabited by multi-cultured ethnic people and is very important place for the studies related to traditional medicine.

Nepal is well-known for rich cultural heritage [40] and comprises 125 different multicultural ethnic groups with more than 123 different languages [48]. In Nepal, indigenous knowledge related to uses of different animals as medicine is generally passed verbally from one generation to the next generation. Such knowledge is commonly lost with the demise of knowledgeable person [4, 5, 49, 50]. It is, thus, important to systematically document indigenous knowledge so that it can be saved for the future generations. This study is aimed at collecting and documenting traditional ethnozoological knowledge related to vertebrates from the Chitwan-Annapurna Landscape, central Nepal. Here, we asked following questions: (i) What are different vertebrates that are used in traditional healing practices in the Chitwan-Annapurna Landscape, central Nepal? What are the modes of preparation and administration of vertebrate based traditional medicine? (iii) What major ailment categories are treated by different vertebrates and also what are the most important vertebrate species used against different ailments? (iv) What is the conservation status of each vertebrate that is used as traditional medicine? To answer the above-mentioned questions, we interviewed 204 locals from the Chitwan-Annapurna Landscape, central Nepal.

## Materials and methods

### Study area

**Fig 1:**
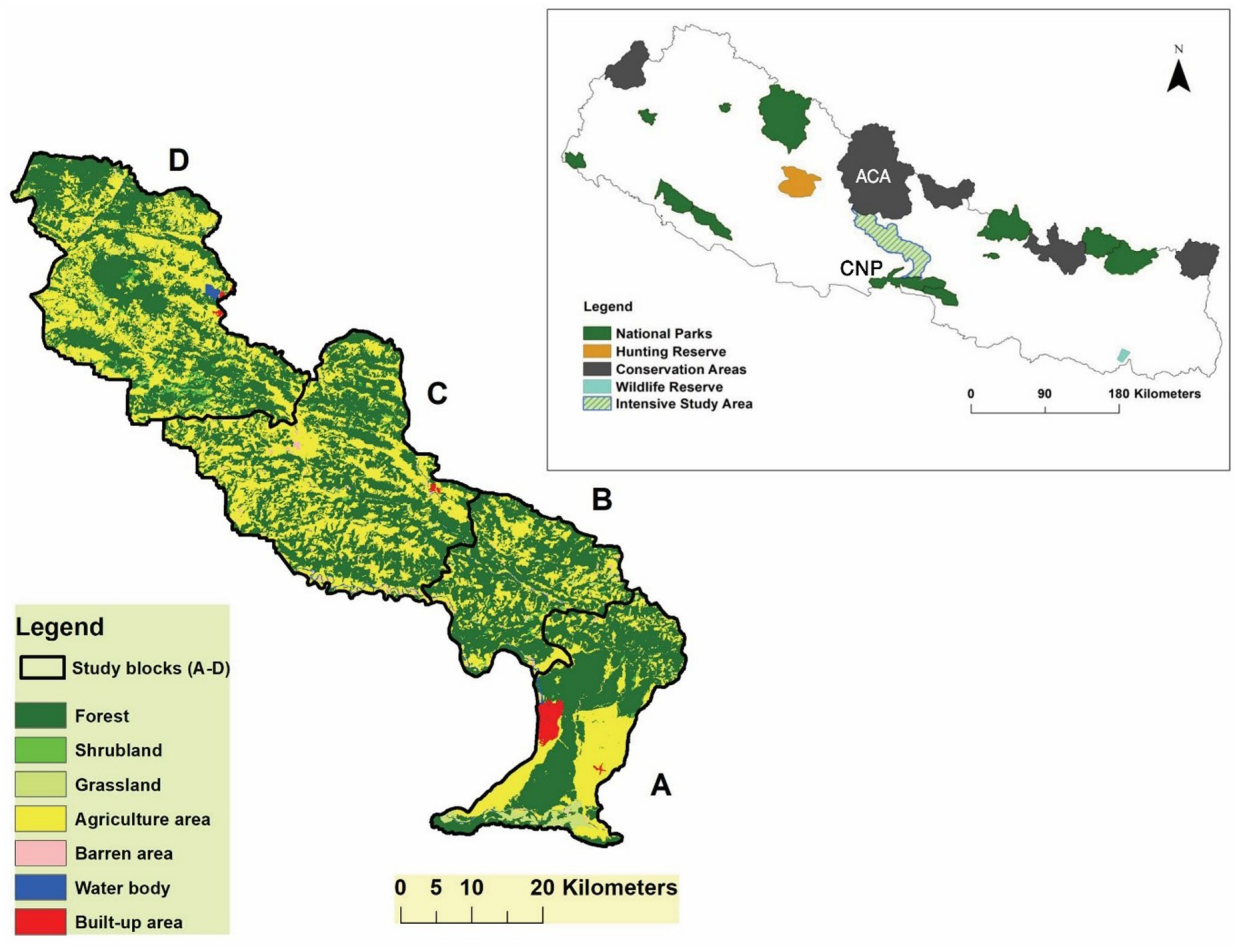
The map shows the intensive study areas which links two biodiversity significant areas: Chitwan National Park (CNP) and Annapurna Conservation Area [52].

The study area is located in the Chitwan-Annapurna Landscape, central Nepal (Fig 1). The region is connected with the Chitwan National Park in the south and Annapurna Conservation Area (ACA) in the north. This landscape is rich in globally outstanding biodiversity, including three Word Wildlife Fund (WWF) Global 200 Eco–regions (Terai–duar Savanna and Grasslands, Himalayan Subtropical Broadleaf Forests, Alpine Shrubs and Meadows) [51], and two Ramsar sites (Beeshazari Lake, Chitwan and Lake Clusters of Pokhara valley, Kaski) [52].

The Chitwan-Annapurna Landscape is a prime habitat for many mammal species such as the tiger, rhinoceros, leopard, clouded leopard, snow leopard, sloth bear, Himalayan black bear, sambar, chital, musk deer, hog deer, Himalayan goral, etc. This landscape is inhabited by diverse communities (23 ethnic groups in Chitwan, 26 ethnic groups in Tanahun, 12 ethnic groups in Kaski) [48]. The most dominant ethnic groups in the study area are Tharu, Braman/Chhetri, Tamang, Gurung, Bote in Chitwan; Magar, Gurung, Sanyasi, Tamang, Majhi in Tanahun and Gurung, Magar, Braman/Chhetri and Dalit in Kaski district [48]. Nepali language is the official language in the study area. More than 70.1% of people of Chitwan district spoke Nepali as their first language followed by 10.2% Tharu, 4.9% Tamang, 3.7% Chepang, 2.8% Gurung, 1.7% Bhojpuri, 1.6% Magar, 1.6% Newari, 1.1% Darai, 0.6% Maithili and 0.5% Hindi. Likewise, 61.9% of people of Tanahun district spoke Nepali as their primary language followed by 20.8% Magar, 8.3% Gurung, 4.1% Newari, 1.1% Darai, 0.8% Urdu and 0.7% Tamang. Similarly, 78.5% of people of Kaski spoke Nepali as their primary language followed by 11.9% Gurung, 2.2% Magar, 2.2% Newari, 1.5% Tamang, 0.7% Bhojpuri, 0.6% Hindi and 0.5% Maithili [48]. The people followed by the Hindu, Buddhist, Christian and Muslim minorities [48].

### Data collection

For data collection, we divided the whole study area into four different study blocks based on topography, climatic variations and ethnic compositions (Fig 1):

Block A: Barandabhar Corridor Forest and its adjoining settlement areas in Chitwan district, Bharatpur Metropolitan City (Patihani, Gitanagar, Bhojad, Ramnagar, Kabilas and Chaukidanda), Ratnanagar Municipality (Sauraha, Mohana, Tikauli, Panchakanya), Kalika Municipality (Jutpani and Padampur). This study block is located in between 27.59761702°N/84.48943997°E to 27.77122°N/84.49986°E. This area has tropical and sub-tropical types of climate having an average maximum and minimum temperature (1989–2018) were 30.86°C and 17.85°C, respectively. The average annual rainfall and relative humidity of Chitwan district (Rampur station number 902, elevation 189m asl) were 1980.34mm and 76.65%, respectively [53, 54].
Block B: Community and National forests and its adjoining settlement areas in lower parts of Tanahun district (27.77611502°N/84.435171°E to 27.92545°N/84.32756667°E), Devghat Rural Municipality (Gaighat, Kaphaldada and Mude), Aanbookhaireni Rural Municipality (Saranghat and Deurali), Bandipur Rural Municipality (Khaharetar, Dharampani and Bandipur), Byas Municipality (Keshavtar, Rumsi, Nayagaun). The average maximum and minimum temperature (1989–2018) were 29.31°C and 17.10°C, respectively. The average annual rainfall and average relative humidity were 2238.98mm and 74.29%, respectively (Khairenitar station number 815, elevation 515m asl) [53, 54].
Block C: Community and National forests and its adjoining settlement areas in upper part of Tanahun district, Rishing Rural Municipality (Manpur, Dhaap, Pipalbot, Chalisegaun), Mygde Rural Municipality (Chhang, Fulbari, Tharpu, Mulabari), Bhimad Municipality (Rishing Patan, Bhimad), Suklagandaki Municipality (Firfire, Therpek, Raipur, Taxar). The geographic location of this block is 27.91741102°N/84.08539603°E to 28.082906°N/83.98566697°E. The average maximum and minimum temperature (1989–2018) were 27.03°C and 15.69°C, respectively. Similarly, the average annual rainfall and relative humidity were 2999.7mm and 62.29% (Pokhara Airport station number 804, elevation 827m asl) [53, 54].
Block D: Panchase area and lower Annapurna Conservation Area, Pokhara Metropolitan City (Nirmalpokhari, Bharatpokhari, Sidhane, Panchase, Pumdibhumdi), Annapurna Rural Municipality (Bhadaure, Tamagi, Landruk and Ghandruk). This study block is located from 28.12748°N/84.04162°E to 28.38574°N/83.79772°E. The average maximum and minimum temperature (1989–2018) were 20.74°C and 12.03°C, respectively. Likewise, the average annual rainfall and relative humidity were 5480.19mm and 81.34% (Lumle station number 814, elevation 1738m asl) [53, 54].

Participatory Rural Appraisal (PRA) method was employed to collect ethno-medicinal data on uses of animals (mainly vertebrates) [49, 55–57]. In this method, we used sets of questions related to the use of animals as ethnomedicine and discussed their local status and medicinal properties. During data collection, we used the photographs of the vertebrates. Local healers, medicinal practitioners, teachers and social workers were involved in the PRA. We also considered a fair gender composition of the group. PRA provided information on the local status and medicinal uses of animals which further helped for the modification of questionnaires including related information as they suggested before starting the formal interview.

For our study, we followed the ethical guidelines of the International Society of Ethnobiology [58]. Personal consent was taken from each respondent prior to a formal interview. To carry out studies in the Chitwan-Annapurna Landscape, we obtained the permission from the Department of National Parks and Wildlife Conservation (Permission letter number 3372), Chitwan National Park (Permission letter number 2723), Division Forest Offices of Chitwan (Permission letter number 2723), Tanahun (Permission letter number 749), Kaski (Permission letter number 200) districts and Annapurna Conservation Area Project (Permission letter number 66).

Open ended and semi-structured questionnaires were used to collect information from the local people [6, 21, 59]. The questionnaires contained background information, household profiles, use of animals and animal parts for medicine and sanitary issues in Nepali language and later translated into an English language (S1 and S2 Files). The respondents were chosen randomly, but were well representatives from different ethnic groups, geographic locations, age, sex, profession, and education levels [10, 45, 60]. In total, 204 respondents were interviewed during 2018–2019. The respondents were interviewed only once. We used photographs and images of different vertebrates when conducting interviews. The detailed information, including local name of the animals, parts used, methods of preparation and mode of administration were recorded. The geographic coordinates of each respondents’ house were taken by using Global Positioning System (GPS) (S1 Table).

Latin names and classification of the animals were obtained from different literature [61–67]. We also recorded the conservation status of each animal by using the above-mentioned literature and IUCN Red data book [68].

Based on the information obtained from informants in the study area, all the reported human related ailments were grouped into 11 categories: cardiovascular problem, dental problem, dermatological problem, ear, nose and throat problem, gastro-intestinal problem, musculoskeletal problem, neurological problem, ophthalmological problem, reproductive problems, respiratory problem and others (fever and headache). In addition to this, we also recorded the veterinary and the agriculture uses (Table 1).

**Table 1:** List of ailments grouped into different categories.

SN	Ailment categories	Biomedical terms	Nepali name
1	Cardiovascular problem	Anemia	Rakta alpata
		Malaria	Aulo jaro
		Snake bite	Sarpa le tokeko
2	Dental problem	Gum bleeding	Dant bata ragat aune (Harsa rog)
3	Musculoskeletal problem	Rheumatism	Bath rog
		Muscular pain and cramp	Masu tuteko/ Dukheko
		Backbone pain	Dhad dukheko
		Arthritis	Haddi khiyeko
		Strength	Baliyo
		Energy	Sakti
		Protein deficiency	Protein ko kami
4	Reproductive problem	Menstrual problem	Mahinabari ma pida, Kharabi
		Sexual performance	Yaunsakti badaune
		Low sperms	Sukrakit kami hunu
		Infertile	Banjo pan
		Hermaphroditism	Napusakata
		Delivery pain	Prasab pida
		Uterine bleeding	Patheghar bata ragat bagnu
5	Ear, Nose and Throat problem	Ear ache	Kan dukheko
		Speech	Boli ma samashya
		Heart disease	Mutu dukheko
6	Respiratory problem	Asthma	Dam
		Hiccups	Hikka hikka hunu
		Cough	Khoki lageko
		Tuberculosis	Kshyarog
		Pneumonia	Nimoniya
		Cold	Chiso lageko
7	Neurological problem	Anxiety	Chinta rog
		Will power	Ichhasakti
		Mental illness	Manasik rogi
		Epilepsy	Chhare rog
		Neurovascular	Nasa sambandi rog
		Ghost	Bhut lageko
		Tetanus	Danustankar
		Rabies	Rebij
		Paralysis	Pyaralaisis
8	Dermatological problem	Wound	Ghau lageko
		Pimples	Dandiphor
		Burning	Poleko, Dadheko
		Marks of old wounds	Purano ghau ko khat
		Facial spots	Anuhar ma kalo thopla aune
		Skin disease	Chhala ko rog
		Scabies	Luto
		Ring worm	Daad
		Loss of hair	Raun jharne
		Allergy	Chilaune rog
		Measles	Dadura ayeko
		Cracks of soles	Paitala phutne
9	Gastro-intestinal problem	Poisoning	Bish lageko, Khana ma kharabi
		Nausea	Wakwaki lagnu
		Ulcer	Andra ma ghau hune
		Endogenous wind	Bayu, Ganogola
		Stomach pain	Pet dukheko
		Gastritis	Amlapitta
		Constipation	Kabjiyat
		Piles	Pile
		Vomiting	Ulti hune
		Dysentery	Aaun pareko
		Jaundice	Kamalpitta, Pahele rog
10	Ophthalmological problem	Poor vision	Drishti alpata
11	Others	Headache	Tauko dukheko
		Fever	Jaro aayeko
12	Veterinary and agriculture use	Insecticides	Kitnasak
		Wounds on cattle	Gai lai ghau bhayema
		Mouth and foot disease	Khoret

### Data analysis

#### Informant consensus factor (ICF)

The agreement on the use of animals in the ailment categories between the respondents in the study area was assessed with the informant consensus factor (ICF) [69, 70].

The informant consensus factor (ICF) for ailment category was calculated as:

$$ICF=\frac{\text{Nur}-\text{Nt}}{\text{Nur}\;-1}$$

Where, Nur is the number of use-reports in each ailment category and Nt is the total number of taxa used in each ailment category by all informants.

In each case if an animal was mentioned by an informant as ‘used’, then we considered to be one ‘use-report.’ If one informant reported an animal as a treatment for more than one ailment in the same category, we considered it as one use-report [71]. Thus, an animal species could be listed in several ailment categories of indigenous uses, but in terms of use-reports, each animal species was considered only once per informant in a single ailment category as described by Amiguet et al. [71].

The ICF ranges from 0 to 1, where high values (close to 1) are obtained when only one or a few animal species are reported to be used by a high proportion of informants to treat a particular ailment meaning that there is a narrow well-defined group of animal species used to cure a particular ailment category and/or that information is exchanged between informants. On the other hand, low ICF values (close to zero) indicate that informants disagree over which animal to use due to random choosing or lack of exchange of information about the use among informants [72].

#### Fidelity level

To determine the most frequently used animal species for treating a particular ailment category of the local people of the study area, we calculated the fidelity level [73]. For each species *s* and each ailment category c, we calculated the value FL_sc_ using the following formula:

$${\text{FL}}_{\text{sc}}(\text{\%})=\frac{{\text{Np}}_{\text{sc}}}{\text{Ns}}\times 100$$

where *Np*_*sc*_ is the number of use-reports cited for a given animal species *s* for a particular ailment category *c* and *N*_*s*_ is the total number of use-reports cited for any given species *s*. The animal species with the highest FL_sc_ value is considered the most preferred species for ailment category *c*.

#### Use value

The relative importance of an animal species used as medicine in the study areas was calculated with the help of the use value for the species *s* [74]:

$${\text{UV}}_{\text{s}\;}=\frac{\sum {\text{U}}_{\text{s}}}{{\text{N}}_{\text{s}}}$$

where U_s_ is the number of use-reports cited by each informant for a given animal species *s* and N is the total number of informants interviewed for a given animal species *s*. Use values are high when there are many use-reports for an animal and low when there are few reports related to its use.

We used Spearman’s correlation coefficient to determine the correlations between FL value and UV values for each animal species because data were not normally distributed.

Multivariate test was used to see the effects of age, gender, elevation, sampled blocks and education (literate/illiterate) and distance from the nearest city on composition of animal species recorded in our study. We used Canonical Correspondence Analysis (CCA) because the gradient length was long enough (2.86) with presence and absence data [75]. Analysis was carried out by using Canoco 5.12 [76]. The significance of the predictors was tested by using Monte Carlo permutation test (n = 499). In CCA, we down weighted the rare species to reduce their effect on the results and significant variables were selected by using a forward selection process.

## Results and discussion

### Demographic details of informants

A total of 204 informants (70 female and 134 male individuals, aged between 18 and 82 years) participated in the study. They belong to different castes (12) and communities (eight of Indo-Aryan or Tibeto-Burman language speaking groups). A large number of respondents were in between 50 to 59 years (n = 53). This clearly showed that ethno-medicinal knowledge was higher in aged groups than in young groups. The main reason of little knowledge among younger generation might be due to the growing number of hospital facilities, migration of people to urban areas and abroad for study and employment and also the influences of mixed cultures due to cross-cultural communications or settlements in new areas [2, 20, 28, 33, 50, 77].

Most people living in the villages have strong belief in the traditional healing system and traditional medicine. The male and female ratio may indicate the dominance of the males versus females among ethno-medicinal practitioners. Similar trends were reported in other studies [5, 41, 43, 77–79] (Table 2). About 43% of the respondents were farmers and healers who had broad knowledge of ethno-medicine. There were 38% of the respondents with basic level and 26% of them with secondary level education. Locally popular traditional healers and wizard doctors were involved in the focus group discussion.

**Table 2:** Demographic profile of the respondents.

Questionnaires (n = 204)	Block A	Block B	Block C	Block D	Total	Percentage
No of household interviewed	52	45	58	49	204	100
**Occupation**						
**Farmer**	19	21	28	19	87	43
**Student**	4	6	4	3	17	8
**Teacher**	8	4	8	5	25	12
**Social worker**	5	5	7	5	22	11
**Government employee**	7	5	3	1	16	8
**Hotel owner**	2	0	0	6	8	4
**Business**	7	4	8	10	29	14
**Gender**						
**Female**	15	12	27	16	70	34
**Male**	37	33	31	33	134	66
**Education status**						
**Illiterate**	4	5	6	9	24	12
**Literate**	14	18	28	17	77	38
**Secondary**	16	11	13	14	54	26
**Intermediate**	9	6	7	4	26	13
**University**	9	5	4	5	23	11
**Ethnic group**						
**Dalit**	1	6	2	6	15	7
**Gurung**	8	4	10	34	56	27
**Magar**	2	16	28	0	46	23
**Newar**	0	3	7	0	10	5
**Tamang**	3	5	0	4	12	6
**Darai**	4	2	0	0	6	3
**Sanyasi**	0	2	7	0	9	4
**Braman/Chhetri**	5	5	4	3	17	8
**Gharti**	0	0	0	2	2	1
**Tharu**	18	0	0	0	18	9
**Mushahar**	6	0	0	0	6	3
**Bote**	5	2	0	0	7	3
**Age group (year)**						
**15–19**	3	3	2	1	9	4
**20–29**	3	3	4	4	14	7
**30–39**	8	6	4	7	25	12
**40–49**	12	10	11	8	41	20
**50–59**	12	14	18	9	53	26
**60–69**	10	6	14	11	41	20
**70–79**	2	3	5	7	17	8
**Above 80**	2	0	0	2	4	2

### Faunal diversity and uses

**Fig 2:**
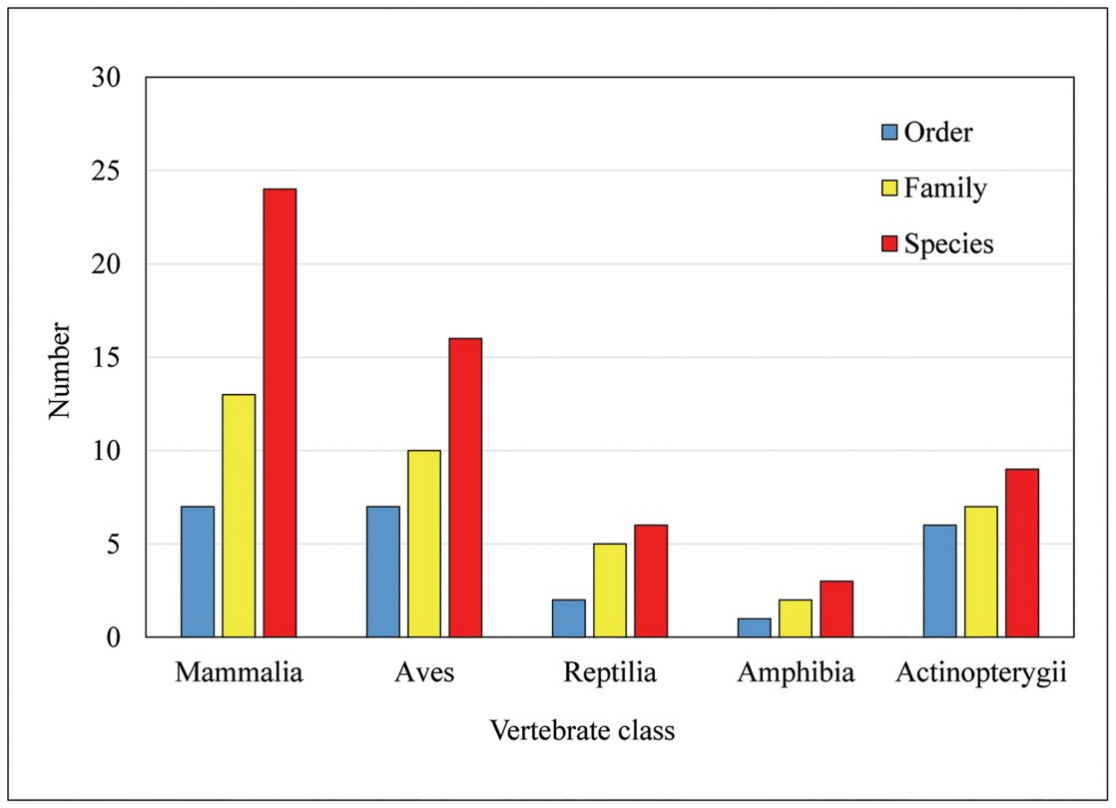
Taxonomic groups of vertebrates used in ethno-medicinal practices among different ethnic communities of the Chitwan-Annapurna Landscape.

The present study revealed the use of 58 animal species belonging to 23 orders, 37 families and 53 genera to cure 62 human and three veterinary ailments (Table 3). Out of all animals, 53 animal species were wild and five were domesticated (Table 3). The order, family, scientific names, English names, Nepali names, IUCN category, use value, parts used and uses are presented in Table 3. Among 58 vertebrates, 24 species (41%) were Mammalia, 16 Aves (28%), 6 Reptilia (1%), 3 Amphibia (0.5%) and 9 Actinopterygii (1.5%) (Fig 2). Out of all animals, only one animal (*Ptyas mucosa*) has poisonous property (Table 3). The use of threatened wild animals as medicine (Table 3) is quite concerning as they might undergo extinction.

**Table 3:** Medicinal uses of vertebrates and their body parts in traditional medicine by the people inhabiting in mid-hills in Nepal.

SN	Family	Scientific Name	English names	Nepali names	IUCN category	UV	Parts used	Uses	Similar use references
**Class: Mammalia**									
**Order: Carnivora**									
1	Canidae	*Canis aureus* Linnaeus, 1758	Golden jackal (W)	Shyal	LC	0.03	Meat, blood, fat	Cooked meat with oat and pea is used for the people suffering from paralysis; wine prepared from the meat is considered as good for people suffering from rheumatism; fresh or cooked blood is believed to good for asthma; massage from the fat or oil will be relief from muscular pain and cramp.	[14, 16, 83, 91]
2	Canidae	*Canis lupus familiaris* Linnaeus, 1758	Black dog (D)	Kalo Kukur		-	Scat	The paste of the old dry scat of the black dog is considered as the good for poisoning.	
3	Felidae	*Panthera pardus* (Linnaeus, 1758)	Leopard (W)	Chituwa	VU	0.04	Meat, Skin, Fat, bone, hair	Cooked meat is used to retain the sexual performance; ghost will not enter into the home, if they have a piece of leopard skin; massage from the fat of leopard, provide relief from back bone pain and arthritis; the ash of the hair is mixed with mustard oil and used in old wounds, help for curing; the soup of the bone is considered as aphrodisiac in nature. **Veterinary and agriculture use-** the dry skin is rubbed and the paste is used in the cattle suffering from mouth and foot disease.	[4, 15, 16]
4	Felidae	*Felis chaus* Schreber, 1777	Jungle cat (W)	Ban Biralo	LC	0.25	Meat	The whole body is unskinned and boiled to make soup and given to the patients of arthritis and poor vision.	
5	Felidae	*Panthera tigris* (Linnaeus, 1758)	Tiger (W)	Baag	EN	0.23	Teeth, brain, blood, skin, meat, fecal matter	The paste of the teeth of tiger is considered as good for rabies, asthma; lotion of the brain is suggested to use in face for pimples and raw brain is also prescribed to eat to remove laziness; blood of tiger is used for strength and develop willpower; paste of dry skin and hair is prescribed in mental illness; cooked meat of tiger is good for nausea and malaria suffering person; dry ash of scat is mixed with black powder, black salt and honey and prescribed to eat for the treatment of burning, piles, epilepsy, ulcer and malaria.	[15, 16, 83]
6	Ursidae	*Ursus thibetanus* G. [Baron] Cuvier, 1823	Asiatic black bear (W)	Kalo bhalu	VU	0.04	Gall bladder, claws	The gallbladder of the bear is cooked with rice or wheat and made dry. Such dry grains are given to the patients of malaria and Jaundice for a week; the claw is rubbed and made a fine paste and used in skin to remove the marks of old wounds.	[15, 16]
7	Ursidae	*Melursus ursinus* (Shaw, 1791)	Sloth bear (W)	Rukh bhalu	VU	0.10	Gall bladder, meat, claws	Dry gall bladder is prescribed to remedy from cold, improve eye sights, and control fever; soup of meat of bear help to stop endogenous wind to arrest convulsion; bear meat is valued as sexualpotency and health booster; the claw is rubbed and made a fine paste and used in skin to remove the marks of old wounds.	[15]
**Order: Cetartiodactyla**									
8	Bovidae	*Naemorhedus goral* (Hardwicke, 1825)	Himalayan goral (W)	Ghoral	NT	0.04	Horn, hoops, meat	The horn of the goral is rubbed and made a fine paste and used in the navel region for curing the stomach pain; the hoops are rubbed and the paste is used to remove the black spots from the face, the cooked meat is used to promote strength and virility.	
9	Bovidae	*Ovis aries* Linnaeus, 1758	Sheep (D)	Bhendo		0.02	Ghee, milk	The massage from the ghee of sheep during muscular cramp is considered as good; the milk of the sheep is mixed with long pepper (*Piper longum* L.) and given to the person suffering from stomach pain.	[4, 15, 42]
10	Bovidae	*Bos taurus* Linnaeus, 1758	Cattle (D)	Gai		0.02	Urine, milk, ghee,	Urine of cow help to control the skin disease while applying on the skin twice a day for a week; urine also help to relief from gastritis while drinking half tea glass early in the morning before meal; Milk of cow helps to promote strength and virility; massage by ghee of cow gets relief from muscular and joints pain. **Veterinary and agriculture use-** urine of cattle is used in the crops for killing the harmful insects likewise, droppings of cow helps to reduce the insects in the crops.	[4, 15, 16]
11	Bovidae	*Bubalus bubalis bubalis* (Linnaeus, 1758)	Buffalo (D)	Bhaisi		0.03	Meat, fecal matter	Meat is used to promote strength and virility; Dry dung is burnt and mixed with mustard oil and applied to cure measles and scabies.	[4, 91]
12	Cervidae	*Rusa unicolor* (Kerr, 1792)	Sambar (W)	Jarayo	VU	0.05	Horn, Meat	The antler is rubbed and make a fine paste and used on the face is help to make the fair face, past of the antler is used to cure old ring worm; the paste is also used around the large wound is help to reduce the rash of wound; cooked meat is used to promote strength and virility.	[16, 42]
13	Cervidae	*Muntiacus vaginalis* (Boddaert, 1785)	Northern red muntjac (W)	Rate, Rato mirga	LC	0.05	Meat, horn	Cooked meat helps to relief the person suffering from heart disease; the antler is rubbed with water and used as ear drops during earache.	[4, 15, 91]
14	Cervidae	*Axis axis* (Erxleben, 1777)	Chital (W)	Chital/Harin	LC	0.06	Antler, Meat	The antler is rubbed and make a fine paste and used on the face is help to make the clear face; the paste is also used around the large wound to reduce the rash; the cooked meat is used to promote strength and virility.	[4, 15, 91]
15	Suidae	*Sus scrofa* Linnaeus, 1758	Wild boar (W)	Bandel	LC	0.04	Meat	The cooked meat is used to promote strength and virility; the soup of dry meat [92] is provided to relief the patient suffering from epilepsy.	[4, 15]
16	Suidae	*Sus domesticus* Erxleben, 1777	Pig (D)	Sungur		0.08	Gall bladder, Fat	Gall bladder is boiled and mixed with honey and black salt and given the person suffering from asthma for a month; the melted fat of pig is used in the face as lotion to cure pimples.	[4, 15]
**Order: Chiroptera**									
17	Rhinolophidae	*Rhinolopus* sp.	Bat (W)	Chamero		-	Meat	The cooked meat of bat is good for asthma; the meat soup is given to the patients twice in a day for one months to cure from tuberculosis.	[16, 93]
**Order: Lagomorpha**									
18	Leporidae	*Lepus nigricollis* F. Cuvier, 1823	Indian hare (W)	Kharayo	LC	0.04	Blood, Meat, Hair	Fresh blood of rabbit is given to the patients for drinking for the treatment of asthma; cooked meat of rabbit is given at least 3 days for the treatment of menstrual problems; the ash of the hair is mixed with mustard oil and used in wounds.	[15, 83]
**Order: Perissodactyla**									
19	Rhinocerotidae	*Rhinoceros unicornis* Linnaeus, 1758	Indian Rhinocers (W)	Gaida	VU	0.16	Horn, Meat, Urine	The powder of horn of rhino is advised to use the person suffering from fever, arthritis, anxiety and food poisoning, cooked meat or soup is suggested to eat for the treatment of paralysis and tuberculosis; urine is used as ear drops to cure ear ache.	[4, 16]
**Order: Primates**									
20	Cercopithecidae	*Semnopithecus hector* (Pocock, 1928)	Tarai gray langur (W)	Kalo Bandar	NT	0.09	Meat	Cooked meat is believed to use for the relief of rheumatism, asthma, anemia.	[4, 15]
21	Cercopithecidae	*Macaca assamensis* M’Clelland, 1840	Assam macaque (W)	Pahare Bandar	NT	-	Meat	The meat of the monkey is cooked with small pea and given to patients suffering from Tuberculosis for a month.	
**Order: Rodentia**									
22	Hystricidae	*Hystrix indica* Kerr, 1792	Indian crested porcupine (W)	Dumsi	LC	0.04	Stomach, Meat, Quails, Fecal matter	Stomach and intestine parts are dried (along with oat) and given to people suffering from the asthma; cooked meat is given to the children suffering from cold and stomach pain; wizard doctors use the quails to protect the sick people from ghost; dry fecal matter is grinded well and make a fine paste with honey and given to patients suffering from abdomen pain.	[14]
23	Muridae	*Rattus rattus* (Linnaeus, 1758)	House rat (W)	Muso	LC	-	Meat	The cooked meat is considered as good for increasing sperms of male.	[16]
24	Sciuridae	*Petauista* sp.	Flying squirrel (W)	Rukh Lokharke	LC	0.09	Meat, Fat	The un-skinned body of flying squirrel is kept into the mustard oil and used for massage; the hair on the head will reappear when the oil of the squirrel is used on the head for a month.	
**Class: Aves**									
**Order: Ciconiiformes**									
25	Ciconiidae	*Leptoptilos javanicus* (Horsfield, 1821)	Lesser adjutant (W)	Garud	VU	-	Claws, Meat	The paste of claws of stork is applied on the place of snake bite and considered as extraction of poisons from bite; hot soup of meat is prescribed to eat for the patient of malaria for a month.	
**Order: Charadriiformes**									
26	Charadriidae	*Vanellus indicus* (Boddaert, 1783)	Red-wattled lapwing (W)	Hutitaun	LC	0.04	Egg	Egg is given to the person suffering from gum bleeding and piles.	[4, 36]
**Order: Columbiformes**									
27	Columbidae	*Treron sphenurus* (Vigors, 1832)	Wedge-tailed green-pigeon (W)	Haleso	LC	0.02	Meat	The soup of meat is given the paralysis suffering person for a month; cooked meat is considered as good for cold suffering person.	[4]
28	Columbidae	*Columba livia* Gmelin, 1789	Rock dove (W)	Parewa	LC	0.14	Meat, Fecal matter	Cooked meat is given to the patients of paralysis; dry fecal matter is applied as a paste with mustard oil to treat boils and blisters.	[14, 36, 91]
29	Columbidae	*Streptopelia orientalis* (Latham, 1790)	Oriental turtle-dove (W)	Dhukur	LC	-	Meat	Soup of the meat is prescribed protection from cold.	[42]
**Order: Galliformes**									
30	Phasianidae	*Arborophila torqueola* (Valenciennes, 1826)	Hill partridage (W)	Pyura	LC	-	Meat	The soup of meat is considered as good for sexual potency and infertile male and female.	
31	Phasianidae	*Lophura leucomelanos* (Latham, 1790)	Kaliz pheasant (W)	Kaliz	LC	-	Meat	Cooked meat is used to promote strength and virility for child and child bearing mothers.	[4, 42]
32	Phasianidae	*Francolinus francolinus* (Linnaeus, 1766)	Black francolin (W)	Titra	LC	0.02	Egg, Meat	Boiled egg is given to the anemia suffering women; cooked meat is used to promote strength and virility.	[42]
33	Phasianidae	*Gallus gallus* (Linnaeus, 1758)	Red jungle fowl (W)	Ban Kukhura	LC	0.09	Fat, meat	It will get relief when the fat/oil of the Red jungle fowl is used in burning wounds; cooked meat and soup is used to promote strength and virility.	[4, 42]
34	Phasianidae	*Pavo cristatus* Linnaeus, 1758	Common pea fowl (W)	Mayur	LC	0.08	Feather, Meat, Egg	Ash of feather is mixed with coconut oil and prescribed to use for the patients suffering from headache, hiccups and vomiting; cooked meat is prescribed to use for energy, and protect from cold; boiled egg is suggested to use for gum bleeding.	[4, 15, 36]
**Order: Passeriformes**									
35	Passeridae	*Passer domesticus* (Linnaeus, 1758)	House sparrow (W)	Bhagera	LC	0.10	Meat	The paste of the meat is used on the anus of the baby to control constipation; fume is applied on the whole body for controlling allergy; the head of the sparrow is used for increasing sexual potency.	[4, 14, 36]
36	Sturnidae	*Acridotheres fuscus* (Wagler, 1827)	Jungle myna	Sarau	LC	-	Meat	The soup prepared from the meat of common myna with black powder, black salt is considered as good for coughing and pneumonia.	[14, 36]
37	Corvidae	*Corvus splendens* Vieillot, 1817	House crow (W)	Kag	LC	-	Blood	The raw blood is applied to treat wounds of the skin and crakes of the sole of feet.	
**Order: Pelecaniformes**									
38	Ardeidae	*Bubulcus ibis* (Linnaeus, 1758)	Cattle egret (W)	Bakulla	LC	-	Meat	Cooked meat of heron is prescribed during gum bleeding and protection from hot.	
**Order: Psittaciformes**									
39	Psittacidae	*Psittacula krameri* (Scopoli, 1769)	Rose-ringed parakeet (W)	Suga	LC	0.04	Meat	Meat of parrot is considered as good for the production of speech in child and it also helps for sexual performance to adults.	[83]
**Order: Strigiformes**									
40	Tytonidae	*Tyto alba* (Scopoli, 1769)	Common barn-owl (W)	Huchil	LC	-	Meat	Meat is boiled and eaten with black salt for treatment of dysentery.	
**Class: Reptilia**									
**Order: Chelonia**									
41	Chelonidae	*Nilssonia hurum* (Gray, 1830)	Indian peacock softshell turtle (W)	Kachhuwa	VU	0.04	Meat, Shell	Raw meat is used to cure from piles; the shell is rubbed and the paste is given for uterine bleeding cases.	
**Order: Squamata**									
42	Agamidae	*Calotes versicolor* (Daudin, 1802)	Common garden lizard (W)	Chheparo	LC	-	Meat	Meat cooked and eat for the treatment of Jaundice. **Veterinary and agriculture use-** the whole body is boiled in mustard oil and then used to heal wounds on cattle’s body.	[14, 18]
43	Colubridae	*Ptyas mucosa* (Linnaeus, 1758)	Ratlle snake (W)	Dhaman	LC	0.07	Fat	Fat is melted and applied on affected part of burning; melted fat is applied for massage in backbone pain.	
44	Gekkonidae	*Hemidactylus flaviviridis* Rüppell, 1835	Northern house gecko (W)	Mausuli	LC	-	Fat	The whole body is boiled with Mustard oil and the oil is used to heal eczema.	
45	Varanidae	*Varanus bengalensis* (Daudin, 1802)	Bengal monitor lizard (W)	Bhainse Gohoro	LC	-	Meat	The boil meat is suggested to eat for the treatment of ringworm.	[36]
46	Varanidae	*Varanus flavescens* (Gray, 1827)	Golden monitor lizard (W)	Sun Gohoro	LC	0.07	Meat, fat, skin	Boiled meat is suggested to eat for arthritis; cooked meat is suggested to use against rheumatism; fat is melted and applied in burning place and scabies, belt made by dry skin is used during backbone pain.	[4, 15]
**Class: Amphibia**									
**Order: Anura**									
47	Bufonidae	*Duttaphrynus himalayanus* (Günther, 1864)	Common toad (W)	Khasre Bhyaguto	LC	-	Meat	Meat is boiled and given to the patients of heart disease mixing with honey.	[14, 36]
48	Dicroglossidae	*Hoplobatrachus tigerinus* (Daudin, 1802)	Tiger frog (W)	Pahelo Pawa	LC	0.06	Fat, Meat	Oil of tiger frog is used in old wound, cooked meat is given to the pregnant women and other anemic attack persons for energy.	
49	Dicroglossidae	*Hoplobatrachus rugulosus* (Wiegmann, 1834)	Black frog (W)	Kalo Pawa	LC	0.09	Legs, Meat	Dry legs of black frog are hanged on the neck small kids, so that they can suck easily. It is regarded as the legs provides the more energy than breast feeding to child; cooked meat of frog is given to people suffering from stomach pain and suffering from cold.	[14, 36]
**Class: Actinopterygii**									
**Order: Anguilliformes**									
50	Anguillidae	*Anguilla bengalensis* (Gray, 1831)	Indian mottled eel (W)	Raj Bam	NT	0.05	Meat	Cooked meat is prescribed to eat for controlling anemia and neurovascular disorders; fish soup is prescribed to eat for controlling asthma; fish oil and soup of meat is advised to use for the treatment of muscular pain and cramp.	[14, 36]
**Order: Cypriniformes**									
51	Balitoridae	*Acanthocobitis botia* (Hamilton, 1822)	Striped loach (W)	Garela Machha	LC	-	Meat	Cooked meat is used to promote strength and virility, sexual performance and control hermaphroditism.	
52	Cyprinidae	*Tor putitora* (Hamilton, 1822)	Mahasheer (W)	Sahar	EN	0.04	Gall bladder, Blood, Fat	Gall bladder of fish is dried with oats, wheat etc. and given to the patients suffering from fever; blood is used in the sore wounds in the foot; fish oil has more protein and supply to children for growth and mental development. **Veterinary and agriculture use-** fresh blood of the Sahar is used to the animals suffering from mouth and foot disease.	[14, 36]
53	Cyprinidae	*Schizothorax richardsonii* (Gray, 1832)	Asla (W)	Asala	VU	-	Meat	Use to promote strength for pregnant women.	[15]
54	Cyprinidae	*Pethia conchonius* (Hamilton, 1822)	Rosy barb (W)	Sidhre	LC	-	Meat	Fish is cooked with black piper and holy basil (*Ocimum tenuiflorum*) and the paste is used for the treatment of pneumonia.	[4, 36, 83]
**Order: Osteoglossiformes**									
55	Notopteridae	*Notopterus notopterus* (Pallas, 1769)	Grey feather back (W)	Patala Machha	LC	0.10	Meat	The fish is burned and cooked with mustard oil, black salt, black piper and prescribed to eat for the relief during delivery pain; fish is boiled with black piper, black salt and holy basil (*Ocimum tenuiflorum)* and given to eat during stomach pain	
**Order: Perciformes**									
56	Anabantidae	*Anabas testudineus* (Bloch, 1792)	Climbing perch (W)	Kabai	DD	-	Meat	Head portion of the fish, long pepper (*Piper longum*) and chilly are boiled together and prescribed to eat during menstrual problems.	[14, 36, 83]
**Order: Synbranchiformes**									
57	Synbranchidae	*Monopterus cuchia* (Hamilton, 1822)	Gangetic mudeel (W)	Chuche Bam	LC	0.01	Meat, blood	Boiled meat is prescribed to eat to get relief from muscular pain; raw blood is consumed for the treatment of anemia.	[4, 36, 83]
**Order: Siluriformes**									
58	Siluridae	*Wallago attu* (Bloch & Schneider, 1801)	Cat fish (W)	Buhari	NT	0.05714	Gall bladder, Meat	Boiled bile is prescribed to eat for the treatment of tetanus, cooked meat is prescribed to promote strength and virility.	[36, 83]

Where, W = Wild, D = Domestic, EN = Endangered, VU = Venerable, NT = Near Threatened, DD = Data Deficient, LC = Least Concern, UV = Use value.

Local people and poachers are generally the greatest threats to wildlife in the world [21, 28, 49] and this was also true for our study. Mammals were considered as the most important vertebrate group that were highly used for medicine. Rural people believed that wild mammals are the sources of protein as well as other essential supplementary foods and medicines [3, 80]. Similarly, it has been shown that vertebrates are used for more than 232 traditional animal therapeutic remedies for human and animal health [15], and food [41]. The practice of using animals in traditional medicine was high in the mid hills and mountainous region than in low land in Chitwan (Table 1). The vertebrate animals such as *Schizothorax* spp., *Tor putitora*, *Hoplobatrachus* spp., *Varanus* spp., *Gallus* sp., *Columba livia*, *Lepus* sp., *Ovis* sp., *Bubalus bubalis bubalis* have both food and medicinal values [36, 81, 82]. Animals such as *Felis chaus*, *Canis lupus familiaris*, *Melursus ursinus*, *Corvus splendens*, *Hemidactylus flaviviridis*, *Duttaphrynus himalayanus* have only medicinal values and are used either cooked or raw. The ethno-medicinal uses of *Canis lupus familiaris*, *Felis chaus*, *Naemorhedus goral*, *Macaca assamensis*, *Leptoptilos javanicus*, *Arborophila torqueola*, *Corvus splendens*, *Bubulcus ibis*, *Tyto alba*, *Nilssonia hurum*, *Ptyas mucosa*, *Hemidactylus flaviviridis*, *Hoplobatrachus tigerinus*, *Acanthocobitis botia* and *Notopterus notopterus* have not been reported prior to our study. The scats, meat, fur, fat, claws, and the blood of these animals were used to treat different ailments such as gum bleeding, sole crakes, tuberculosis, cardiovascular disorders, dysentery, backbone pain, and skin diseases (Table 3).

Generally, the meat of *Canis aureus* was cooked for treating paralysis and preparation of wine for treating rheumatism. Similar practices were also reported by Lohani [41] in the Tamang community of Sindhupalchowk and Poudel and Singh [83] who reported use of meat and fat of Golden jackal for treating rheumatism in the Darai community of Chitwan, Nepal. Similarly, Rai and Singh [42] reported such practices in the Rai community of Bhojpur. Bones and meat of the animals are full of calcium, protein and phosphorous hence, soup of the bone and meat of animals is given to the person suffering from the muscle spasm, cramp, bone fracture and arthritis (Table 3). A similar uses were also reported by Lohani [41] in the Tamang community, Nepal; Vijayakumar et al. [5] in Kerela adjoining areas of Mt. Abu Wildlife sanctuary, India; Nijman and Shepherd [49] in Kyaiktiyo, Myanmar.

The locals in our study area used animal flesh and fat for the treatments of different skin diseases, burning, inflammation, increasing sexual performance, arthritis and poor vision (Table 3). Omega-3 fatty acid present in the fat and meat reduces inflammations and may useful in neurological disorder, thrombosis, and aging [27, 84]. Similarly, milk and butter (ghee) of *Bos taurus* and *Ovis aries* were used to treat muscular cramp and pain, weakness, strength, and virility. Ghee of *Ovis aries* is very useful for burning. Milk and butter have high contents of proteins, lipids, vitamins, and minerals which help to strength the body, relief in joint pains, muscular cramp, increase the strength, and virility [4, 85, 86]. Cooked meat of *Columba livia* is given to the patients with paralysis whereas dry fecal matter is applied as a paste with mustard oil to treat burns and blisters. Similar to our findings was also reported in different places in Nepal [4, 14, 15, 83] but the fresh blood of pigeon is used for treating paralysis in Rajasthan, India [86].

**Fig 3:**
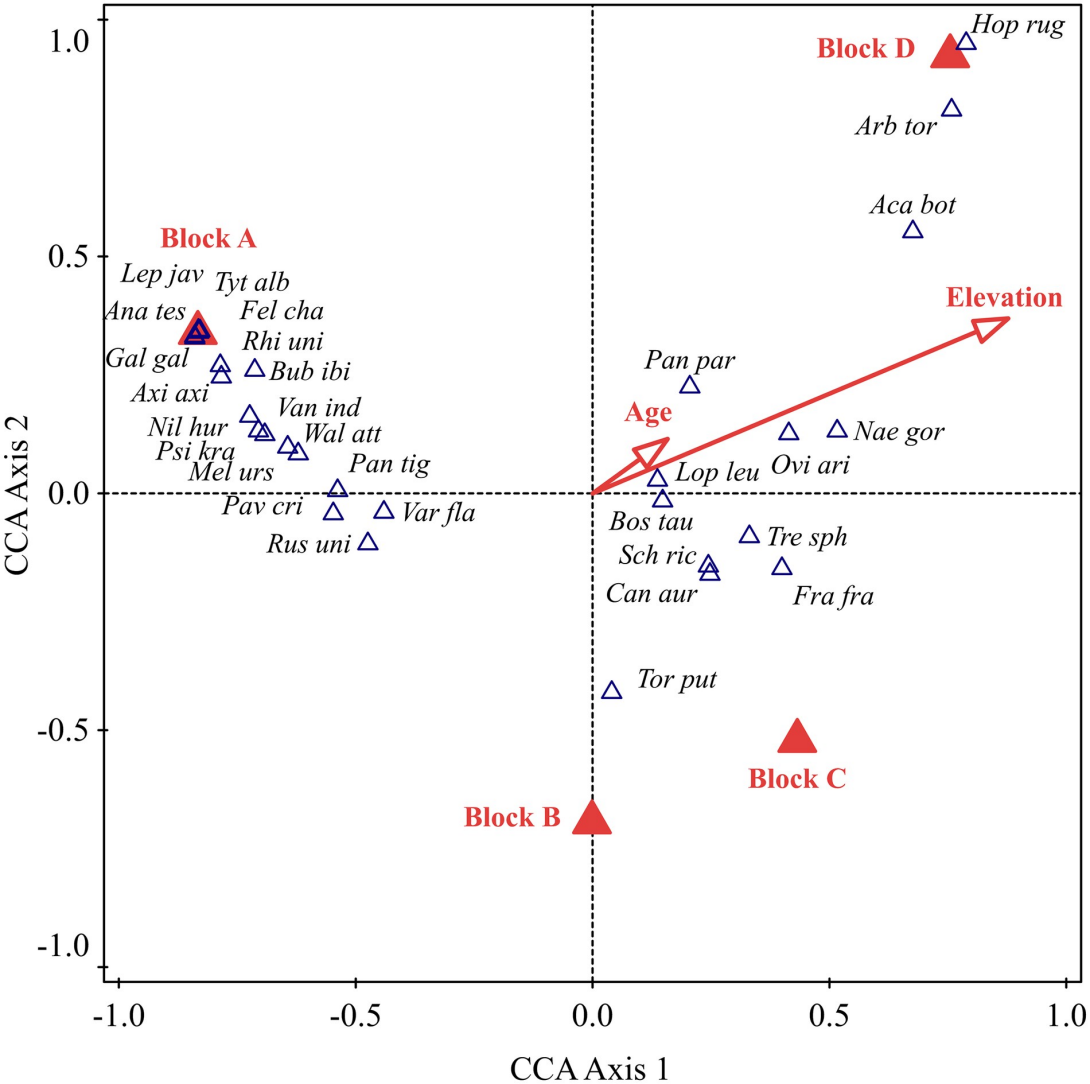
The relationship between the different animal species used for medicine and different environmental factors. The 1^st^ canonical axis explained 16.98% and the 2^nd^ 4.19% of the total variation in the data. Abbreviations: Aca bot-*Acanthocobitis botia*; Ana tes-*Anabas testudineus*; Arb tor-*Arborophila torqueola*; Axi axi-*Axis axis*; Bos tau-*Bos taurus*; Bub ibi-*Bubulcus ibis*; Can aur-*Canis aureus*; Fel cha-*Felis chaus*; Fra fra-*Francolinus francolinus*; Gal gal-*Gallus gallus*; Hop rug-*Hoplobatrachus rugulosus*; Lep jav-*Leptoptilos javanicus*; Lop leu-*Lophura leucomelanos*; Mel urs-*Melursus ursinus*; Nae gor-*Naemorhedus goral*; Nil hur-*Nilssonia hurum*; Ovi ari-*Ovis aries*; Pan par-*Panthera pardus*; Pan tig-*Panthera tigris*; Pav cri-*Pavo cristatus*; Psi kra-*Psittacula krameri*; Rhi uni-*Rhinoceros unicornis*; Rus uni-*Rusa unicolor*; Sch ric-*Schizothorax richardsonii*; Tor put-*Tor putitora*; Tre sph-*Treron sphenurus*; Tyt alb-*Tyto alba*; Van ind-*Vanellus indicus*; Var fla-*Varanus flavescens*; Wal att-*Wallago attu*.

The composition of the animal species recorded for medicinal purposes was affected by sampling blocks, age of informant and elevation of the place where information was collected during our study (p = 0.002 in all factors). It was found that most of the animal species were found in Block A followed by Block D and least were in Block B and C as Block A is located in adjoining areas of the Chitwan National Park and Block D is located inside and around the Annapurna Conservation Area but Block B and C are in human-dominated highly fragmented landscape. In Block A, the species found were *Leptoptilos javanicus*, *Tyto alba*, *Felis chaus*, *Anabas testudineus*, *Gallus gallus*, *Rhinoceros unicornis*, *Bubulcus ibis*, *Axis axis*, *Nilssonia hurum*, *Vanellus indicus*, *Psittacula krameri*, *Wallago attu*, *Melursus ursinus* and *Panthera tigris*. In Block D species recorded were *Hoplobatrachus rugulosus*, *Arborophila torqueola* and *Acanthocobitis botia*. Few species were found in high elevation and they were *Naemorhedus goral*, *Ovis aries* and *Acanthocobitis botia*. Middle-aged people mentioned more about the uses of species than young or too old (Fig 3). Middle-aged people (50 to 60) possess the significant traditional knowledge compared to younger and too old as their long interaction with animal species and they can easily memories the used animals and plants for the treatment of different ailments than too old people [4, 10, 27, 87].

### Important traditional medicines and insecticides for veterinary use

Medicinal fauna from four different animal families belonging to four genera and four species have veterinary importance. Generally, animal body parts and products such as urine, droppings, fat and meat were used. They were used both internally and externally (Table 3). However, most of the people in the study area had little idea and knowledge about veterinary and agricultural use of vertebrates. Furthermore, this study also indicated that more than 81% of vertebrate species were used for treating more than one ailment. Similar studies outside Nepal also reported the wide use of animals in ethno-medicinal purposes such as González et al. [88] recorded use of 30 wild vertebrates to treat domestic animals in Spain, Souto et al. [79, 89] reported 11 animals for ethnoveterinary medicine in the semi-arid region of Northeastern Brazil and Gupta et al. [90] reported a total of 11 species of vertebrates for treating various veterinary diseases in India.

The reason using animal products in Nepal is due to existing tradition and nutritional values of animals and/or their products. The people who live near the forest are more familiar with animals, their nutritional value, and medicinal properties as in the case of the Chitwan-Annapurna Landscape.

### Animal parts used

**Fig 4:**
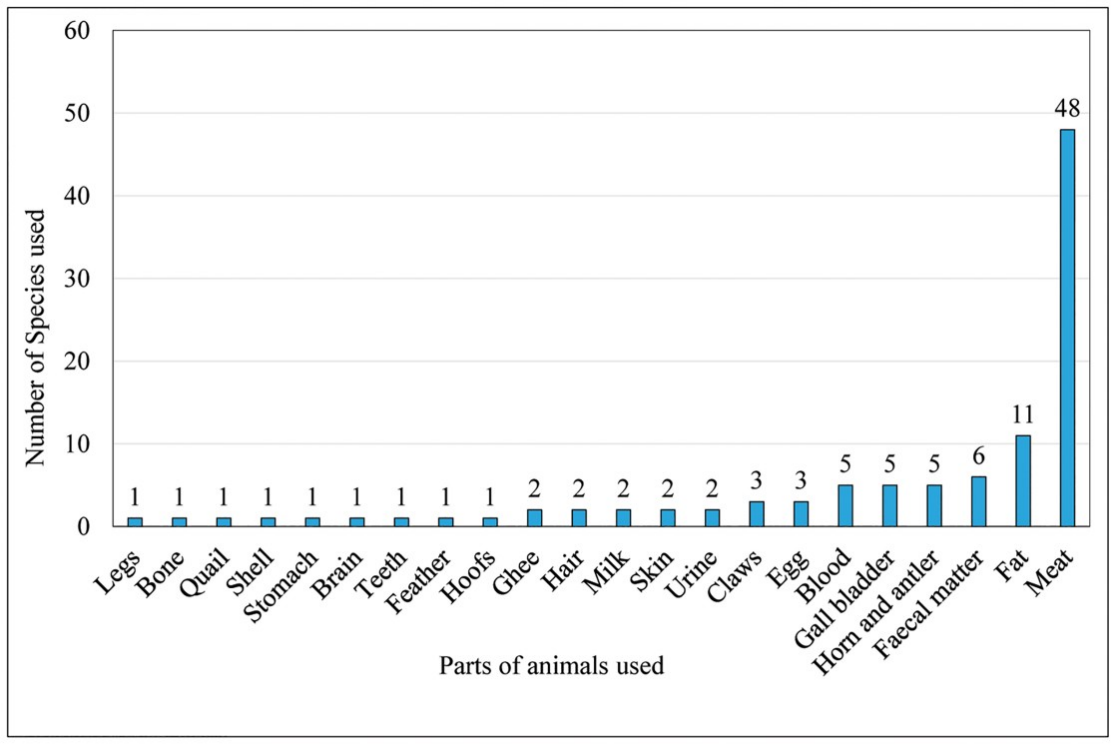
The percentage contribution of body parts of vertebrates used in ethno-medicine.

**Fig 5:**
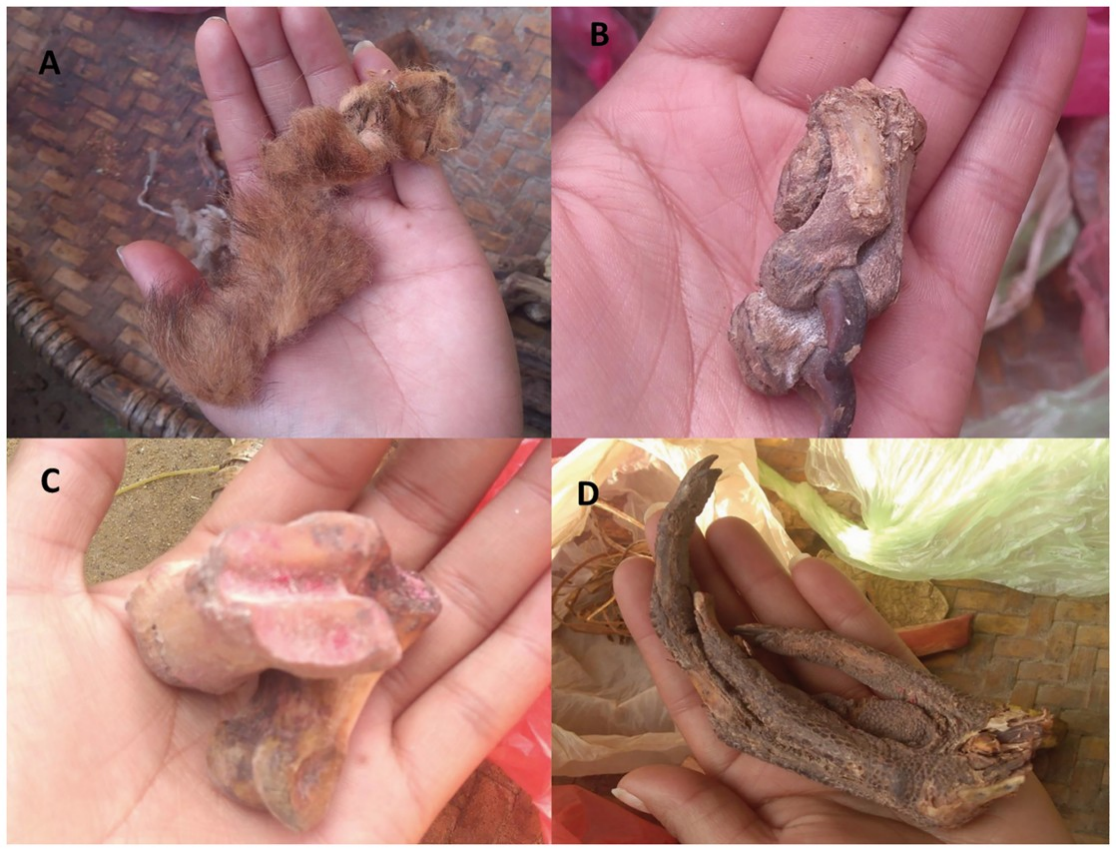
Parts of animals used by local ethnic groups for the treatment of different diseases: A- Skin of Tiger (paste of dry skin and hair is prescribed in mental illness) B- Dry meat of Wild boar (the soup of dry meat (leg) is provided to relief the patient suffering from epilepsy) C- Bone of Leopard (the soup of the bone or paste is considered as aphrodisiac in nature). D- Leg of Lesser adjutant (The paste of claws of stork is applied on the place of snake bite).

**Fig 6:**
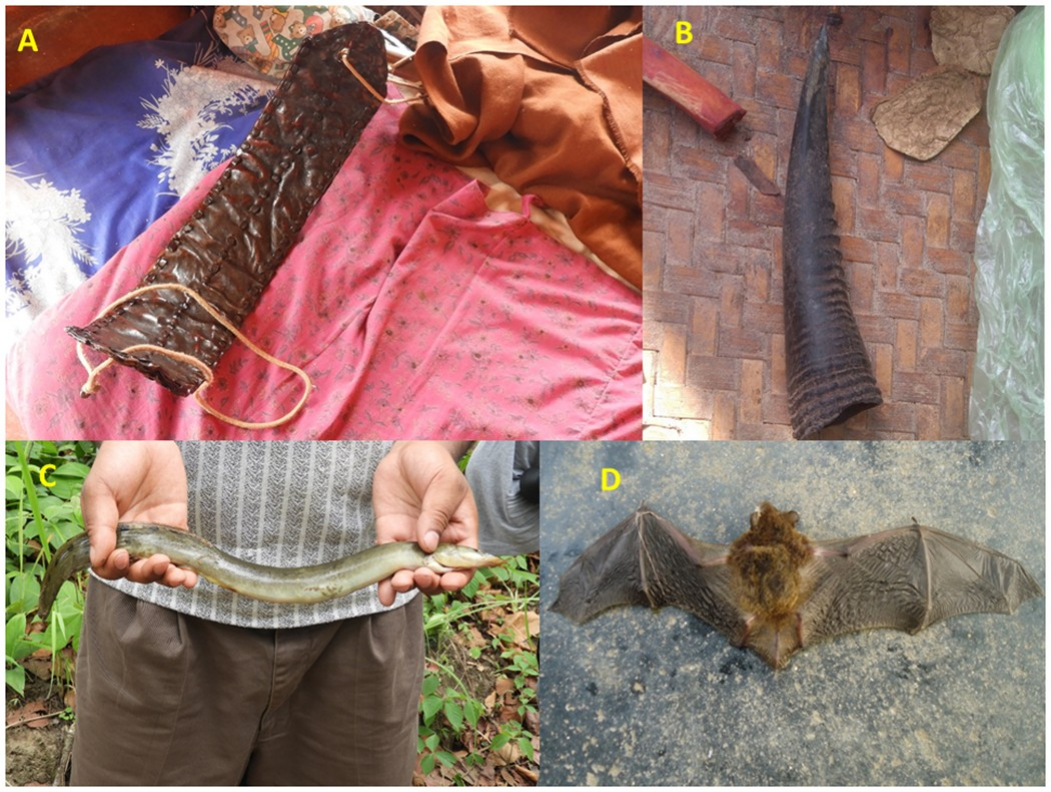
Parts of animals used by local ethnic groups for the treatment of different diseases: A- Belt made by the skin of Monitor lizard (belt made by dry skin is used during backbone pain) B- Horn of Himalayan goral (The horn of the goral is rubbed and made a fine paste and used in the navel region for curing the stomach pain) C- Gangetic mudeel (Boiled meat is prescribed to eat to get relief from muscular pain; raw blood is consumed for the treatment of anemia). D- Bat (The cooked meat of bat is good for asthma, tuberculosis).

The uses of ethno-medicines prepared from various animal body parts and products were also reported in previous studies [3–5, 14, 32, 33, 42, 55, 94]. The animal parts used for treating different ailments were of 22 types. Meat was the most preferred parts (n = 48), possibly because meat has more protein and medical properties, followed by fat (n = 11), fecal matter (n = 6), gallbladder, horn and antler and blood (each n = 5), egg and claws (each n = 3), urine, skin, milk, ghee and hair (each n = 2), hoofs, feathers, teeth, brain, stomach, shell, quail, bone and legs (n = 1) (Figs 4–6). Quave et al. [15] also reported use of the whole animals, milk and milk products, meat/animal flesh, fat, honey, eggs, feces, urine and seminal fluid for different treatments by many ethnic groups of people in Albania, Italy, Spain and Nepal.

### Medicine preparations and their administration

**Fig 7:**
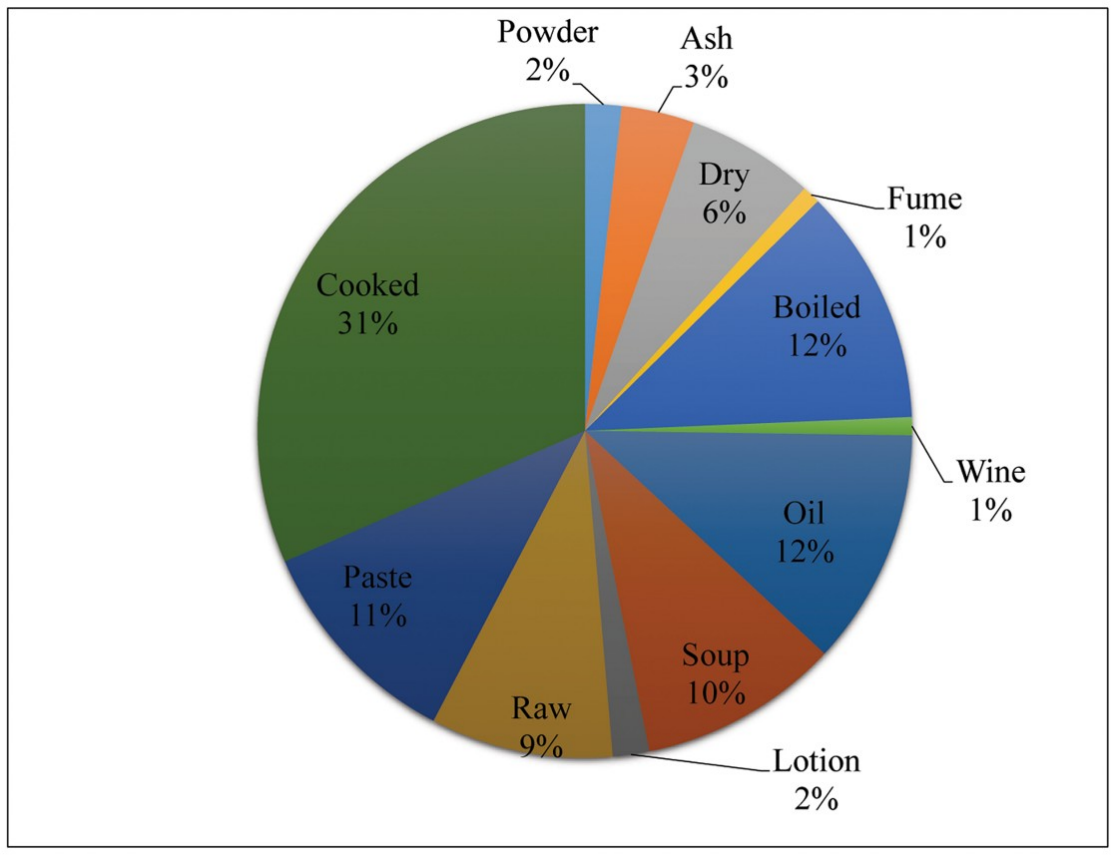
Mode of preparation of medicine from body parts of vertebrates.

The medical remedies were based on many kinds of medicines ranging from a preparation made out of a single animal for a single ailment to the use of animals in combination (Table 3). There were 12 types of preparations used in the study area. Cooked meat was commonly practiced (31%) followed by boiled body parts and oil (each 12%), paste (11%), soup (10%), raw meat (9%), dry (6%), ash (3%), powder and lotion (each 2%) and fume and wind (each 1%) (Fig 7).

The questions related to sanitary/cleanliness issues while using the animals and animal products as traditional medicines and food were asked to the respondents. The results showed that 73% of the respondents were aware about sanitary issues while using the animals and animal products for traditional medicines and they used traditional methods such as sun drying, smoking, boiling, cooking, roasting, and washing before use. But 36.3% of the respondents had the knowledge about the transfer of diseases such as rabies, influenza, plague and tuberculosis from animals to human. Similarly, 47.5% of the respondents responded about the transformation of parasites such as roundworm, tapeworm and liver fluke from animals to human. Consumption practices of raw body parts of animals are common for curing diseases in many ethnic groups at global level [2, 5, 28, 55, 94, 95]. However, consumption of raw meat may increase the risks of transmitting different types of parasites and diseases to humans [4, 94]. Zoonotic diseases can be transmitted by direct contact with animals and also by using animal products as food and medicine. The care should be taken while using raw flesh or blood as a traditional medicine because major zoonotic diseases such as tuberculosis, rabies and influenza can easily be transmitted from animals to human beings [96–98]. It has been found that avian influenza (Influenza A, H1N1) viruses were transmitted from wild birds to human and have caused devastating effects [99].

The most common mode of administration of medicine is oral (67%) followed by topical application (30%) and drop (4%). Topical use is an important way of remedy for musculo-skeletal problems like muscular pain, fractures, rheumatisms and arthritis. Such modes of administration were found in studies from Korea [94], in India [5] in Brazil [33, 80, 100] and also in Nepal [4, 83].

### Informant consensus factor, fidelity level and use value

The results of the informant consensus factor (ICF) calculation showed that the value of our study ranges from 0.833 to 0.974. Cardiovascular and dental problems have the highest ICF value 0.974, with cardiovascular problems having 351 use-reports for 10 animal species (*Panthera tigris*, *Ursus thibetanus*, *Muntiacus vaginalis*, *Semnopithecus hector*, *Leptoptilos javanicus*, *Francolinus francolinus*, *Bufo bufo*, *Hoplobatrachus tigerinus*, *Anguilla bengalensis*, *Monopterus cuchia*) and dental problems having 77 use-reports for 3 animal species (*Vanellus indicus*, *Pavo cristatus*, *Bubulcus ibis*). It is followed by musculoskeletal problems (ICF = 0.973, 926 use-reports, 46 species). The least agreement between the informants was observed for animals used to cure ophthalmological uses with ICF value 0.833 with 7 use-reports for 2 animal species (Table 4). The low ICF value might be due to lack of communication for the treatment of such ailments among the people of different cultures, different localities and ethnicities of the study area. Local people believed that there was no any side effects while using these ethnomedicines.

**Table 4:** Categories of ailments and informant consensus factor (ICF) for these categories.

Ailment categories	Number of use-reports	Number of taxa (Nt)	Informant consensus factor (ICF)
Cardiovascular problem	351	10	0.974
Dental problem	77	3	0.974
Musculoskeletal problem	926	26	0.973
Reproductive problem	355	12	0.969
Ear, Nose and Throat problem	63	3	0.968
Respiratory problem	452	16	0.967
Neurological problem	262	12	0.958
Others	47	3	0.957
Dermatological problem	369	22	0.943
Gastro-intestinal problem	263	17	0.939
Ophthalmological problem	7	2	0.833
**Total**	**3172**	126*	

*A taxon may be reported in more than one ailment category.

When selecting the most preferred animal species for each ailment category, we took the highest FL (%) in each category of ailment (Table 5). *Leptoptilos javanicus* and *Duttaphrynus himalayanus* for cardiovascular ailments, *Bubulcus ibis* for dental problem, *Corvus splendens*, *Hemidactylus flaviviridis* and *Varanus bengalensis* for dermatological problem, *Canis lupus familiaris*, *Tyto alba* and *Calotes versicolor* for gastro-intestinal problem, *Lophura leucomelanos* for musculo-skeletal problem, *Rattus rattus*, *Arborophila torqueola*, *Acanthocobitis botia* and *Anabas testudineus* for reproductive problem, *Rhinolopus* spp., *Macaca assamensis*, *Streptopelia orientalis*, *Acridotheres fuscus*, *Schizothorax richardsonii* and *Pethia conchonius* for respiratory problem has the highest FL (100% each) and *Felis chaus* has the lowest (20%) for ophthalmological problem purposes. The 100% animals indicate that healers and local respondents used that animals for the treatment of the same disease. It implied that well-known species were used more than the little-known species to cure the disease or disorders [57, 94].

**Table 5:** Most frequently used animal(s) for different ailment categories based on the highest FL (%) in each ailment category.

Ailments	Animal	FL (%)
Cardiovascular problem	*Leptoptilos javanicus* (Horsfield, 1821)	100
	*Bufo bufo* (Linnaeus, 1758)	100
Dental problem	*Bubulcus ibis* (Linnaeus, 1758)	100
Dermatological problem	*Corvus splendens* Vieillot, 1817	100
	*Hemidactylus flaviviridis* Rüppell, 1835	100
	*Varanus bengalensis* (Daudin, 1802)	100
Gastro-intestinal problem	*Canis lupus familiaris* Linnaeus, 1758	100
	*Tyto alba* (Scopoli, 1769)	100
	*Calotes versicolor* (Daudin, 1802)	100
Musculoskeletal problem	*Lophura leucomelanos* (Latham, 1790)	100
Reproductive problem	*Rattus rattus* (Linnaeus, 1758)	100
	*Arborophila torqueola* (Valenciennes, 1826)	100
	*Acanthocobitis botia* (Hamilton, 1822)	100
	*Anabas testudineus* (Bloch, 1792)	100
Respiratory problem	*Rhinolopus* spp.	100
	*Macaca assamensis* M’Clelland, 1840	100
	*Streptopelia orientalis* (Latham, 1790)	100
	*Acridotheres fuscus* (Wagler, 1827)	100
	*Schizothorax richardsonii* (Gray, 1832)	100
	*Pethia conchonius* (Hamilton, 1822)	100
Ear, Nose and Throat problem	*Psittacula krameri* (Scopoli, 1769)	75.6
Neurological problem	*Wallago attu* (Bloch & Schneider, 1801)	60
Others	*Tor putitora* (Hamilton, 1822)	56.9
Ophthalmological problem	*Felis chaus* Schreber, 1777	20

The most commonly used species was *Felis chaus* (UV = 0.25) with 3 use-reports by 10 informants. It was followed by *Panthera tigris* (UV = 0.23) with 6 use-reports by 57 informants, *Rhinoceros unicornis* (UV = 0.16) with 6 use-reports by 43 informants and *Columba livia* (UV = 0.14) with 3 use-reports by 22 informants (Table 3).

The correlation between the highest fidelity level (%) in ailment categories and animal use value was not significant (Spearman’s correlation test: r^2^ = 0.038, p = 0.209) indicating that the animals systematically used for a specific ailment category are not necessarily those used commonly in the region. Although animals with high FL or UV are the most preferred species in study sites (Tables 4 and 5), animals with low FL or UV should not be neglected as failing to mention them could increase the risk of the gradual disappearance of the knowledge.

### Conservation status

**Fig 8:**
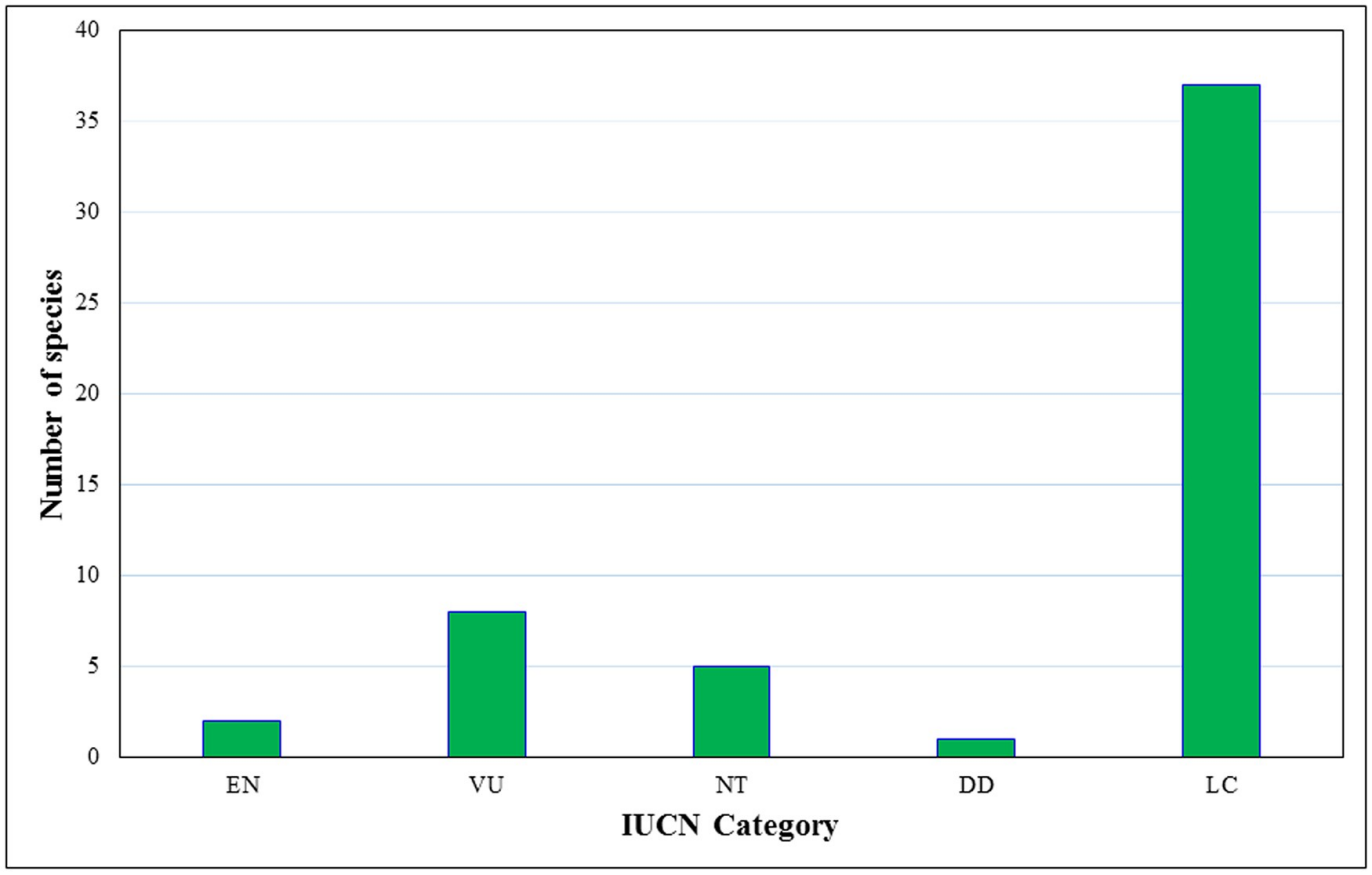
Conservation status of wild vertebrate species used in ethnomedicine in the Chitwan-Annapurna Landscape, Nepal (according to IUCN Red List, 2019).

Due to lack of the modern medical facilities and belief on the traditional healing system, people in the study area are forced to use animals and their body parts for curing various ailments. Besides, superstitions and mythologies were very popular among the ethnic groups that may also play vital roles in using the animals. Therefore, they intentionally or unintentionally kill animals for ethno-medicinal uses which might increase threats to wildlife including many globally threatened species. Out of total, ten animals were Globally Threatened (2 Endangered, 8 Vulnerable), five were Near Threatened, one was Data Deficient and 37 Least Concern species according to the IUCN Red List [68] (Table 3, Fig 8). The Government of Nepal implemented National Parks and Wildlife Conservation (NPWC) Act 1973 and Forest Act 1993 to protect wildlife and their habitats in Nepal. These laws strictly prohibited the hunting and killing of wildlife. There is no permission of killing the wildlife listed in CITES for food, medicine, and their trade. However, sometimes these laws malfunction due to local religious norms and cultural beliefs. Therefore, to protect the wild animals, the local traditional communities should be aware of the alternative method of treatment systems such as the use of medicinal plants instead of animal products. Study of Jaroli et al. [32] found that among 24 identified animals used by Garasiya people in adjoining areas of Mount Abu Wildlife Sanctuary, India, 16 animals (including elephant, tiger, sambar, Himalayan black bear) are included in IUCN Red List. If the people didn’t think about the alternative methods of treatment systems, it will bring great problems on wildlife conservation [28, 79].

Extinction risk is very high for the vertebrates as compared to invertebrates [101, 102]. Vertebrates are more prone to habitat loss, exploitation, poaching and illegal trade. Besides, ethno-medicinal uses of vertebrates offer the reasons for increasing threats to their conservation. Therefore, this study is mainly devoted to vertebrates. The ethnic communities and local healers need search for other treatment options such as plants instead of animals. For example, fruits of *Rhus javanica* can be used instead of cooked blood of *Canis aureus* when treating asthma [57, 103, 104]. Seed oil of *Impatiens scabrida* can help to relieve body pain instead of fat of *Panthera pardus* [57, 105–107]. Similarly, powder prepared from the roots of *Heracleum wallichii* used for treating stomach problems to substitute meat of *Naemorhedus goral*, *Hystrix indica* and *Nanorana liebigii* [108, 109]. This study suggests that there is enough space for a researcher to document alternatives of the vertebrates for ethno-medicinal value.

## Conclusions

The study is the first effort to document primary data of the ethno-medicinal knowledge about the use of vertebrates by the local people of the Chitwan-Annapurna Landscape. Ethno-medicinal knowledge about vertebrates and their body parts and products play the vital role in conservation and consumption of those species. A total of 58 species of vertebrates used for the treatment of 62 human ailments which were grouped into 11 categories. Mammals contributed the highest number among them in ethno-medicine. This study also indicated that more than 76% of vertebrate species were found to be used for the treatment of more than one ailment. The most commonly used species was *Felis chaus* and cardiovascular and dental problems had the highest ICF value. Traditional knowledge was more in middle-aged people than young and too old people because of long experiences of utilizing nature and can easily memories the uses of animals and their products. Therefore, our study concluded that there is a necessity for documentation of detailed knowledge about the status and specific use-values of vertebrates as well as the transfer of knowledge from seniors to the youths for sustainable ethno-medicine in living with nature places like the Chitwan-Annapurna Landscape, central Nepal. This empirical knowledge described in this study will help in the preparation of conservation planning to control the hunting of threatened wildlife. Furthermore, ethnic people should consider the alternative options such as the use of commonly found medicinal plants and other inorganic salts or compounds for the treatments of ailments. Any future economic gains obtained using indigenous knowledge should be shared with local communities to safeguard their intellectual property rights.

## Supporting information

S1 Table Global Positioning System (GPS)- latitude and longitude coordinates of each respondent who was interviewed during data collection. (PDF)

S1 File Open ended and semi-structured questionnaire used to record the detail information on ethnozoology (in Nepali language). (PDF)

S2 File Open ended and semi-structured questionnaire used to record the detail information on ethnozoology (in English language). (PDF)
