# Supplementary material for: Ethno-medicinal uses of vertebrates in the Chitwan-Annapurna Landscape, central Nepal
Source: PLoS One. 2020 Oct 30;15(10):e0240555. doi: 10.1371/journal.pone.0240555 (PMC7598503; doi:10.1371/journal.pone.0240555)
Supplement: S1 Table — (PDF) [file pone.0240555.s001.pdf]

S1 Table. Global Positioning System (GPS)- latitude and longitude coordinates of each respondent who was interviewed during data collection.

SN	Location	Longitude	Latitude	Elevation	Block	Ethnic group	Age	Sex	Education	Occupation
1001	Badreni	84.48944	27.59762	213	A	Mushahar	28	F	Intermediate	Social worker
1002	Badreni	84.48643	27.59343	213	A	Mushahar	31	M	Iliterate	Farmer
1003	Mohona	84.49418	27.61206	215	A	Darai	38	M	Secondary	Teacher
1004	Mohona	84.48550	27.61110	214	A	Mushahar	48	M	Iliterate	Farmer
1005	Mohona	84.48501	27.60867	214	A	Bote	18	M	Secondary	Student
1006	Tikauli	84.47930	27.62927	205	A	Tharu	53	F	Literate	Farmer
1007	Tikauli	84.47912	27.62864	205	A	Bote	54	M	Iliterate	Social worker
1008	Tikauli	84.47903	27.62803	204	A	Tharu	49	M	Intermediate	Teacher
1009	Padampur	84.35989	27.57685	217	A	Tharu	51	M	Secondary	Hotel owner
1010	Padampur	84.49466	27.68178	253	A	Tharu	58	F	Secondary	Business
1011	Padampur	84.49363	27.66802	251	A	Tharu	35	M	Intermediate	Teacher
1012	Padampur	84.49846	27.66897	212	A	Darai	57	M	Secondary	Hotel owner
1013	Padampur	84.49578	27.67306	214	A	Tharu	68	M	Secondary	Farmer
1014	Padampur	84.49563	27.67692	216	A	Mushahar	41	M	Iliterate	Social worker
1015	Haripur	84.53404	27.74252	220	A	Tharu	67	M	Secondary	Farmer
1016	Haripur	84.53131	27.73989	221	A	Tharu	51	M	Intermediate	Teacher
1017	Haripur	84.52667	27.73808	223	A	Tharu	50	M	Secondary	Business
1018	Haripur	84.52098	27.73614	372	A	Darai	25	F	University	Student
1019	Haripur	84.52005	27.73679	355	A	Mushahar	44	M	Literate	Business
1020	Gairibari	84.56510	27.72891	343	A	Bote	38	M	University	Teacher
1021	Gairibari	84.56569	27.73005	311	A	Tamang	17	M	Secondary	Student
1022	Gairibari	84.56659	27.73849	312	A	Bote	66	M	Literate	Farmer
1023	Gairibari	84.56600	27.73462	325	A	Bote	67	M	Literate	Farmer
1024	Gairibari	84.56732	27.74393	330	A	Tharu	82	M	Literate	Farmer
1025	Kabilas	84.46449	27.77624	366	A	Mushahar	73	M	Literate	Farmer
1026	Kabilas	84.47603	27.75776	408	A	Gurung	55	F	University	Teacher
1027	Kabilas	84.42711	27.66039	332	A	Gurung	42	F	Secondary	Social worker
1028	Kabilas	84.49986	27.77122	402	A	Gurung	32	M	Literate	Farmer
1029	Gauri ganj	84.42339	27.62513	235	A	Braman/Chhetri	57	F	University	Business
1030	Ghatghai	84.37676	27.56981	220	A	Tharu	39	M	University	Business
1031	Ghatghai	84.37325	27.57284	221	A	Tharu	47	F	Literate	Farmer
1032	Ghatghai	84.36395	27.57155	221	A	Braman/Chhetri	55	F	Secondary	Farmer
1033	Panchkanya	84.49215	27.65455	202	A	Tharu	61	M	Intermediate	Gov. employee
1034	Panchkanya	84.49446	27.65792	204	A	Magar	49	M	Secondary	Teacher
1035	Jirauna	84.49699	27.67050	214	A	Tharu	67	F	University	Gov. employee
1036	Jirauna	84.49777	27.66993	215	A	Tharu	31	M	Literate	Farmer
1037	Jirauna	84.49846	27.66897	215	A	Braman/Chhetri	80	M	Literate	Farmer
1038	Baseni	84.45981	27.69267	201	A	Gurung	41	M	University	Teacher
1039	Baseni	84.45010	27.70479	365	A	Tamang	63	F	Literate	Social worker
1040	Baseni	84.44218	27.70869	224	A	Gurung	53	M	Intermediate	Gov. employee
1041	Devnagar	84.41205	27.61036	222	A	Braman/Chhetri	75		Secondary	Farmer
1042	Devnagar	84.41460	27.60862	224	A	Magar	39	F	Intermediate	Gov. employee
1043	Devnagar	84.40861	27.59949	221	A	Darai	48	M	Literate	Farmer
1044	Ujelinagar	84.39776	27.59168	224	A	Braman/Chhetri	29	M	University	Gov. employee
1045	Ujelinagar	84.39497	27.58984	223	A	Gurung	59	F	Intermediate	Gov. employee
1046	Ujelinagar	84.39113	27.58630	222	A	Gurung	68	M	Literate	Farmer
1047	Bagmara	84.48301	27.61367	214	A	Tharu	44	M	Secondary	Business
1048	Bagmara	84.48479	27.60537	212	A	Tharu	49	F	Secondary	Business
1049	Bagmara	84.48002	27.62546	204	A	Tharu	19	M	Intermediate	Student
1050	Gondrang	84.45658	27.66827	218	A	Gurung	45	M	Literate	Farmer
1051	Gondrang	84.45461	27.66440	217	A	Tamang	67	F	Secondary	Farmer
1052	Gauri ganj	84.44264	27.64445	235	A	Dalit	65	M	University	Gov. employee
2001	Amrite	84.43517	27.77612	363	B	Magar	73	M	Iliterate	Farmer
2002	Amrite	84.43517	27.77612	383	B	Magar	50	M	Literate	Farmer
2003	Baidi area	84.31285	27.85795	347	B	Magar	43	M	Literate	Business
2004	Baidi area	84.30979	27.85692	347	B	Magar	22	F	University	Student
2005	Mudre	84.36730	27.84213	953	B	Magar	45	M	Intermediate	Teacher
2006	Mudre	84.36701	27.84312	966	B	Magar	18	M	secondary	Student

2007	Bagar khola	84.41367	27.85916	1002	B	Magar	17	M	secondary	Student
2008	Bagar khola	84.42183	27.85816	848	B	Magar	78	M	Iliterate	Farmer
2009	Deurali	84.46115	27.88763	805	B	Tamang	32	F	University	Student
2010	Deurali	84.44651	27.87175	628	B	Tamang	67	M	Literate	Farmer
2011	Dharam pani	84.38315	27.88450	1100	B	Tamang	56	M	Literate	Farmer
2012	Dharam pani	84.38363	27.88443	287	B	Magar	38	F	secondary	Social worker
2013	Kamalbari	84.39270	27.88267	335	B	Gurung	57	M	Intermediate	Teacher
2014	Kamalbari	84.39701	27.88447	782	B	Dalit	21	F	secondary	Social worker
2015	Jhar gaun	84.42941	27.90949	788	B	Magar	34	M	Literate	Farmer
2016	Jhar gaun	84.43282	27.91271	912	B	Magar	46	M	Literate	Business
2017	Bahul bhanjyang	84.43561	27.92391	935	B	Magar	67	F	Iliterate	Business
2018	Bahul bhanjyang	84.43252	27.92327	1164	B	Magar	76	M	Iliterate	Farmer
2019	Ghumaune_chimke	84.46410	27.82461	988	B	Magar	25	M	University	Student
2020	Ghumaune_chimke	84.46406	27.82469	996	B	Magar	19	F	secondary	Student
2021	Chimkeshori	84.48183	27.82395	212	B	Tamang	48	F	Intermediate	Teacher
2022	Chimkeshori	84.48263	27.82458	222	B	Tamang	61	M	Literate	Farmer
2023	Devghat-Gaighat	84.46414	27.82490	598	B	Braman/Chhetri	56	M	Literate	Farmer
2024	Devghat-Gaighat	84.46487	27.82343	658	B	Braman/Chhetri	41	F	secondary	Business
2025	Bagandi/Kafaldanda	84.42425	27.80013	230	B	Braman/Chhetri	37	F	secondary	Social worker
2026	Sukhaura	84.35862	27.91560	485	B	Darai	43	M	secondary	Gov. employee
2027	Belthumki	84.35103	27.91867	454	B	Darai	56	M	secondary	Farmer
2028	Chisapani	84.34308	27.92227	725	B	Newar	64	F	Literate	Social worker
2029	Keshab tar	84.34295	27.92362	742	B	Newar	40	M	Intermediate	Teacher
2030	Keshab tar	84.34043	27.92287	776	B	Newar	58	M	secondary	Gov. employee
2031	Rumsi	84.33218	27.92647	781	B	Braman/Chhetri	34	M	University	Gov. employee
2032	Rumsi	84.32807	27.92542	880	B	Bote	52	M	Literate	Farmer
2033	Rumsi	84.32757	27.92545	912	B	Gurung	55	M	Literate	Farmer
2034	Harkapur	84.33883	27.92257	929	B	Gurung	39	F	Literate	Farmer
2035	Thin	84.34070	27.89978	842	B	Gurung	51	M	Literate	Farmer
2036	New gaun	84.34125	27.89342	869	B	Bote	49	M	Literate	Farmer
2037	Pyughar	84.44559	27.83753	868	B	Braman/Chhetri	55	M	Intermediate	Gov. employee
2038	Sarnagghat	84.40728	27.85151	210	B	Dalit	59	M	University	Gov. employee
2039	Khahare tar	84.35173	27.87392	179	B	Dalit	58	F	secondary	Social worker
2040	Ampdanda	84.45518	27.82694	231	B	Dalit	51	M	Literate	Farmer
2041	Ampdanda	84.45501	27.82721	236	B	Dalit	45	M	Iliterate	Farmer
2042	Bagendi	84.44448	27.77704	667	B	Dalit	52	M	Literate	Farmer
2043	Basa mode	84.40554	27.81341	464	B	Magar	45	M	Literate	Farmer
2044	Basa mode	84.40158	27.80650	1132	B	Sanyasi	67	M	Intermediate	Farmer
2045	Sode pul	84.31048	27.91544	206	B	Sanyasi	69	M	Literate	Farmer
3001	Manpur	84.08540	27.91741	891	C	Gurung	55	M	Literate	Farmer
3002	Manpur	84.08064	27.91910	906	C	Gurung	33	F	Secondary	Gov. employee
3003	Manpur	84.07683	27.91855	908	C	Magar	65	M	Literate	Social worker
3004	Archal Bot	84.08177	27.92002	911	C	Magar	19	F	Intermediate	Farmer
3005	Archal Bot	84.08159	27.92096	875	C	Magar	43	F	Secondary	Social worker
3006	Ghiring Deurali	84.08155	27.92090	870	C	Magar	33	M	Literate	Social worker
3007	Ghiring Deurali	84.08414	27.91975	886	C	Magar	55	F	Secondary	Farmer
3008	Ghiring Deurali	84.08448	27.91788	824	C	Magar	45	M	Literate	Business
3009	Mirchulung	84.09331	27.95544	618	C	Magar	75	M	Literate	Business
3010	Ghiring Deurali	84.09548	27.92525	771	C	Newar	51	M	Secondary	Business
3011	Deurali	84.08034	27.91824	831	C	Newar	50	M	University	Teacher
3012	Hatiya Ghiring	84.03074	27.90531	693	C	Magar	23	F	Intermediate	Teacher
3013	Hatiya Ghiring	84.03076	27.90531	693	C	Magar	55	M	Literate	Farmer
3014	Archalchap	84.17513	27.92226	934	C	Newar	68	M	Intermediate	Farmer
3015	Archalchap	84.17928	27.92033	854	C	Newar	35	M	University	Teacher
3016	Duluk danda	84.18625	27.90949	767	C	Dalit	64	M	University	Business
3017	Taluka gaun	84.20932	27.89905	664	C	Dalit	59	F	Secondary	Teacher
3018	Chalise gaun	84.21576	27.89542	649	C	Newar	34	M	Secondary	Farmer
3019	Suke jamun	84.23366	27.90351	1000	C	Magar	53	M	Secondary	Farmer
3020	Char ghare	84.23801	27.90886	989	C	Gurung	72	M	Iliterate	Farmer
3021	Gairapuri	84.26421	27.94266	933	C	Magar	55	F	Literate	Business
3022	Chhap	84.26580	27.94763	918	C	Magar	62	M	Literate	Business
3023	Pipalbot	84.16445	27.92455	1009	C	Magar	58	F	Intermediate	Teacher
3024	Rishing	84.17080	27.92253	968	C	Gurung	17	F	Intermediate	Student

3025	Rishing	84.23101	27.89669	869	C	Newar	22	F	Secondary	Student
3026	Chhang	84.14872	28.00070	568	C	Newar	55	M	Literate	Farmer
3027	Thanggadi	84.12275	27.98010	768	C	Sanyasi	43	M	Literate	Farmer
3028	Thanggadi	84.12283	27.97818	748	C	Sanyasi	45	M	Literate	Farmer
3029	Phulbari gharredi	84.12593	27.97025	408	C	Sanyasi	43	M	Literate	Farmer
3030	Phulbari gharredi	84.12508	27.96870	414	C	Sanyasi	56	M	Secondary	Business
3031	Rishing patan area	84.11488	27.96547	443	C	Sanyasi	55	F	Secondary	Teacher
3032	Rishing patan area	84.11247	27.96532	401	C	Magar	54	F	Secondary	Gov. employee
3033	Vimad area	84.09802	27.97018	437	C	Magar	42	F	Intermediate	Gov. employee
3034	Vimad area	84.08477	27.98207	474	C	Magar	47	F	Literate	Farmer
3035	Magde	84.09410	27.97988	461	C	Magar	53	F	Literate	Farmer
3036	Magde	84.09537	27.97927	460	C	Magar	55	F	Literate	Farmer
3037	Magde	84.09840	27.97813	481	C	Magar	54	F	Literate	Farmer
3038	Samibhanjyang	84.10392	27.98933	822	C	Magar	45	M	Literate	Farmer
3039	Samibhanjyang	84.10725	27.99163	857	C	Magar	40	F	Literate	Farmer
3040	Bahara Chhap	84.10610	27.99188	870	C	Magar	65	M	Secondary	Teacher
3041	Bahara Chhap	84.10702	27.99308	893	C	Sanyasi	64	M	Iliterate	Farmer
3042	Mulabari	84.10728	27.99392	897	C	Sanyasi	54	M	Literate	Farmer
3043	Mulabari	84.11227	27.99590	904	C	Magar	22	M	Intermediate	Student
3044	Mulabari	84.11420	27.99662	868	C	Magar	21	M	University	Student
3045	Lethar	84.11843	27.99618	883	C	Magar	65	F	Literate	Social worker
3046	Manpang	84.16012	28.00733	551	C	Braman/Chhetri	68	F	Literate	Farmer
3047	Manpang	84.16658	28.02459	746	C	Braman/Chhetri	48	F	Literate	Business
3048	Samdanda	84.15256	28.03560	878	C	Braman/Chhetri	49	M	Literate	Farmer
3049	Samdanda	84.14160	28.03697	968	C	Braman/Chhetri	61	F	Secondary	Teacher
3050	Samdanda	84.07134	28.05270	567	C	Gurung	65	M	Literate	Social worker
3051	Firfire	83.98955	28.08267	1220	C	Magar	51	M	Literate	Social worker
3052	Firfire	83.98567	28.08257	1250	C	Magar	74	M	Iliterate	Farmer
3053	Firfire	83.98576	28.08291	1254	C	Magar	70	M	Iliterate	Farmer
3054	Raipur	83.98710	28.04886	614	C	Gurung	61	F	Literate	Social worker
3055	Thamdanda	83.98564	28.05142	685	C	Gurung	63	F	Literate	Farmer
3056	Tharpek	84.08578	28.09279	539	C	Gurung	78	F	Iliterate	Farmer
3057	Tharpek	84.08686	28.09283	567	C	Gurung	68	F	Iliterate	Farmer
3058	Chandi maidan	84.12328	28.09829	1010	C	Gurung	69	F	Literate	Farmer
4001	Tallo Sidhane	84.45137	27.67899	1379	D	Gurung	46	M	Secondary	Hotel owner
4002	Tallo Sidhane	83.82310	28.22753	1458	D	Gurung	52	F	Secondary	Hotel owner
4003	Tallo Sidhane	83.81776	28.22059	1739	D	Gurung	47	M	Literate	Hotel owner
4004	Bhanjyang	83.81135	28.21827	1980	D	Dalit	15	M	Secondary	Student
4005	Bhanjyang	83.81155	28.21768	1985	D	Gurung	32	M	Intermediate	Farmer
4006	Bhanjyang	83.8116	28.2188	1973	D	Gurung	56	F	Intermediate	Teacher
4007	Bhanjyang	83.8144	28.21267	2042	D	Gurung	51	M	Literate	Farmer
4008	Purane (Virbasti)	83.84051	28.24149	1141	D	Gurung	47	F	Secondary	Hotel owner
4009	Purane (Virbasti)	83.82479	28.24052	1518	D	Gurung	33	F	Iliterate	Hotel owner
4010	Upper Sidhane	83.82831	28.22762	1440	D	Braman/Chhetri	26	M	University	Student
4011	Upper Sidhane	83.82801	28.22693	1447	D	Gurung	52	M	Secondary	Hotel owner
4012	Yamagi dada	83.82866	28.22691	1432	D	Braman/Chhetri	56	M	Literate	Farmer
4013	Chattau	83.82853	28.22683	1436	D	Braman/Chhetri	48	M	Literate	Farmer
4014	Duipakhe gaun	83.82582	28.22307	1560	D	Gurung	52	M	Literate	Farmer
4015	Duipakhe gaun	83.82367	28.22222	1617	D	Gurung	52	M	Iliterate	Farmer
4016	Pumdi bhumdi	83.90602	28.20910	1258	D	Dalit	48	M	Iliterate	Business
4017	Pumdi bhumdi	83.90438	28.20869	1273	D	Dalit	58	F	Iliterate	Farmer
4018	Pumdi bhumdi	83.90454	28.20908	1271	D	Dalit	52	F	Iliterate	Farmer
4019	Pumdi bhumdi	83.90752	28.21023	1287	D	Dalit	48	M	Literate	Business
4020	Arthar Dada	83.77307	28.21499	1521	D	Gurung	33	M	Secondary	Farmer
4021	Arthar Dada	83.77240	28.21494	1496	D	Gurung	65	M	Iliterate	Farmer
4022	Arthar Dada	83.77283	28.21479	1501	D	Gurung	45	F	Iliterate	Farmer
4023	Arthar Dada	83.77508	28.21318	1587	D	Gurung	42	M	University	Teacher
4024	Arthar Dada	83.77535	28.21298	1602	D	Gurung	62	M	secondary	Gov. employee
4025	Arthar Dada	83.77550	28.21281	1607	D	Dalit	68	F	Literate	Social worker
4026	Kaule	83.77936	28.21250	1689	D	Tamang	33	F	Literate	Farmer
4027	Bhadaure	83.82017	28.26301	1578	D	Gharti	27	M	Literate	Business
4028	Bhadaure	83.82209	28.26553	1529	D	Gharti	20	M	Intermediate	Student
4029	Bhadaure	83.82517	28.26496	1461	D	Gurung	63	F	Iliterate	Farmer

4030	Chitre	83.76564	28.24437	1604	D	Gurung	65	M	University	Teacher
4031	Odare	83.79136	28.25660	1499	D	Gurung	61	F	Secondary	Business
4032	Ramja	83.74703	28.24152	1552	D	Gurung	60	F	Literate	Social worker
4033	Ramja	83.75087	28.23615	1620	D	Gurung	60	M	University	Teacher
4034	Ghadruk	83.79772	28.38574	1865	D	Gurung	66	M	Literate	Farmer
4035	Ghadruk	83.81476	28.38219	1585	D	Gurung	64	M	University	Teacher
4036	Landruk	83.82610	28.36963	1505	D	Gurung	77	M	Secondary	Social worker
4037	Landruk	83.82572	28.36750	1509	D	Tamang	75	F	Literate	Farmer
4038	Tolka	83.82950	28.35249	1594	D	Gurung	73	F	Literate	Farmer
4039	Tolka	83.82375	28.34599	1632	D	Gurung	39	M	Intermediate	Farmer
4040	Vendi kharka	83.83051	28.34038	1619	D	Gurung	71	M	Secondary	Social worker
4041	Deurali	83.83200	28.33995	1637	D	Gurung	74	M	Literate	Social worker
4042	Australian camp	83.82735	28.30345	1898	D	Gurung	70	F	Literate	Business
4043	Goldanda	83.82075	28.30025	1741	D	Gurung	71	M	Secondary	Business
4044	Bharatpokhari	84.04162	28.12748	1056	D	Gurung	81	M	Literate	Farmer
4045	Bharatpokhari	84.04008	28.11411	922	D	Tamang	38	M	Literate	Business
4046	Nirmal pokhari	84.03130	28.13249	1073	D	Gurung	67	M	Iliterate	Farmer
4047	Ghaderi neta	83.76629	28.20734	1520	D	Gurung	86	M	Secondary	Business
4048	Raldanda	83.76037	28.21717	1362	D	Gurung	25	F	Secondary	Business
4049	Ghatichinna	83.86240	28.23879	765	D	Tamang	33	M	secondary	Business
