# Supplementary material for: Ethno-medicinal uses of vertebrates in the Chitwan-Annapurna Landscape, central Nepal
Source: PLoS One. 2020 Oct 30;15(10):e0240555. doi: 10.1371/journal.pone.0240555 (PMC7598503; doi:10.1371/journal.pone.0240555)
Supplement: S1 File — (PDF) [file pone.0240555.s002.pdf]

S1 File. Open ended and semi-structured questionnaire used to record the detail information on ethnozoology (in Nepali language)

कुन कुन जनावार हरु परामपरागत औषधी बनाउन, कसरी र के रोगका लागि प्रयोग गरिन्छ भन्ने कुरा पत्ता लगाउने उदेश्यले यो प्रश्नाबली तयार गरिएकोछ। यदि यहाँहरुलाई उत्तर दिन इच्छा छैन भने यहाँ स्वतन्त्र हुनुहुन्छ। यस प्रश्नाबलीमा हजुरहरुको नाम उल्लेख गरिनेछैन तसर्थ तपाईंहरुको कुन उत्तर हो भन्नेकुरा कसैलाई थाहा हुँदैन। यो प्रश्नवलीको उत्तर दिन करीब आधा घण्टा लाग्नेछ। यदी कुनै प्रश्नको उत्तर हजुरहरुलाई दिन मन छैन भने बिचैमा छोड्न पाउनुहुनेछ। तपाईंहरुसँग यो अनुसन्धानको बारेमा केही प्रश्नहरु छन भने कुनै पनि समयमा सोध्न सक्नुहुनेछ। तपाईंहरु सँग केही जिज्ञासा छ त?

प्र. हजुरले यो प्रश्नावलीमा सहभागी हुन चाहनुहुन्छ? छ । छैन , छ भने ......

सामान्य जानकारी

स्थलगत अनुसन्धान कर्ता: मिति:

समय: मौसम: घरबाट नजिक को सहर: औसत दुरी:

व्यक्तिगत विवरण

१. जिल्ला: २. नगरपालिका/ गाउँ पालिका: ३. वार्ड नम्बर:
४. गाउँ/टोल :

५. जी पी एस नम्बर: देशान्तर: अक्ष्यांस: उचाई:

६. जाति: 7. उमेर: ८. लिङ्ग (√): महिला /पुरुष /अन्य

९. शैक्षिक योग्यता (√): निरर्क्षेर/ साक्षर/ विद्यालय स्तर /प्रबिनता प्रमाणपत्र तह / बिश्वबिद्यालय तह

१०. पेशा (√): सरकारी जागिर/ जागिर /शिक्षक /कृषक /सामाजिक कार्यकर्ता / विद्यार्थी /व्यापार /होटेल ब्याबसायी अन्य...................

११. औषधिको लागि प्रयोग हुने जनावर तथा तिनको अंगहरु (यस क्षेत्रमा पाउने जनावरको फोटो देखाएर सोध्ने: Shrestha 2008, Shah and Tiwari 2004, Grimette et al. 2016, Baral and Shah 2008)

क्र. स.	जनावारको नाम	स्थानिय नाम	अंगको प्रयोग	रोगको प्रकार	तयार गर्ने तरिका र उपयोग गर्ने बिधी

१२. सरसफाइ सम्बन्धी

क. जनावार र तिनका उत्पादन प्रयोग गर्दा के तपाईंले साबधानी अपनाउनु हुन्छ?, हुन्छ भने कस्तो? ....

ख. तपाईंलाई जनावार तथा तिनको उत्पादन बाट रोग सर्छ भन्ने थाहा छ? छ भने कुन कुन रोग? ....

ग. तपाईंलाई जनावार तथा तिनको उत्पादन बाट परजिबीहरु सर्छ भन्ने थाहा छ? छ भने कुन कुन परजिबीहरु?......

(**प्रश्नावली को अन्त्यमा : प्रश्नहरु को उत्तर दिनु भएकोमा धेरै धेरै धन्यवाद। तपाईं को जानकारी र समयको लागि हामी आभारी छौं।**)

धन्यवाद ।
