# Supplementary material for: Ethno-medicinal uses of vertebrates in the Chitwan-Annapurna Landscape, central Nepal
Source: PLoS One. 2020 Oct 30;15(10):e0240555. doi: 10.1371/journal.pone.0240555 (PMC7598503; doi:10.1371/journal.pone.0240555)
Supplement: S2 File — (PDF) [file pone.0240555.s003.pdf]

S2 File. Open ended and semi-structured questionnaire used to record the detail information on ethnozoology (in English language)

This questionnaire is being given to find out what types of vertebrate animals are used as ethno-medicine. You do not have an answer these questions if you do not want to. We will not write your name on the questionnaire and no one will know which answers are yours. Answering the questions will take about 30 minutes. You can skip any questions you like by saying skip or stop answering anything at any time you prefer. If you have any questions, you can ask me now or after you anything at any time you prefer. Do you have any questions for me right now about the survey?

Would you like to participate in the survey? (If yes, proceed to background information)

Background information

Name of interviewer: Interview date:
Time: Weather:
Name of the nearest market: Average distance:

Household profile

1. District name: _____________2. Municipality/Rural Municipality: ___________

3. Ward no. _________ 4. Village/Area name_______________________

5. GPS coordinate of the house: GPS no: ____

Longitude: ___________ Latitude____________ Altitude (metre) _______

6. Ethnicity of respondent: ___________________________ 7. Age ____________

8. Sex (√): Male/Female/other____________

9. Highest educational qualification of respondent (√):

Illiterate/ Literate/Secondary/Intermediate/University

10. Occupation: Government service or service/ Teacher/Farmers/ Social worker/ Business/ Hotel owner/ if any, please specify____________

11. Use of animals and animal parts for medicine (Photographs shown from: Shrestha 2008, Shah and Tiwari 2004, Grimette et al. 2016, Baral and Shah 2008)

Name of animal	Local name	Parts use	Ailment category	Preparing and using method

12. Sanitary issues

a. Do you apply any precaution to uptake animals and animal products?

If yes, what type of precautions? _________________________

b. Do you know the zoonotic diseases transfer from animals and animal products?

__yes, __no. If yes, list the major diseases you know____________

c. Do you know the transfer of parasites from animals to man, via animal products?

__yes, ___no. If yes, list the major parasites you know____________

(End of interview): Thank you very much for answering these questions for me. I appreciate your ability and time to help gather information.

Thank you
